# The dipeptide prolyl-hydroxyproline promotes cellular homeostasis and lamellipodia-driven motility *via* active β1-integrin in adult tendon cells

**DOI:** 10.1016/j.jbc.2021.100819

**Published:** 2021-05-23

**Authors:** Kentaro Ide, Sanai Takahashi, Keiko Sakai, Yuki Taga, Tomonori Ueno, David Dickens, Rosalind Jenkins, Francesco Falciani, Takako Sasaki, Kazuhiro Ooi, Shuichi Kawashiri, Kazunori Mizuno, Shunji Hattori, Takao Sakai

**Affiliations:** 1 Department of Pharmacology and Therapeutics, MRC Centre for Drug Safety Science, Institute of Systems, Molecular and Integrative Biology, University of Liverpool, Liverpool, UK; 2 Nippi Research Institute of Biomatrix, Toride, Ibaraki, Japan; 3 Department of Biochemistry and Systems Biology, Institute of Systems, Molecular and Integrative Biology, University of Liverpool, Liverpool, UK; 4 Department of Biochemistry, Faculty of Medicine, Oita University, Oita, Japan; 5 Department of Oral and Maxillofacial Surgery, Kanazawa University Graduate School of Medical Science, Kanazawa, Ishikawa, Japan

**Keywords:** collagen-derived peptide, prolyl-hydroxyproline, adult tendon cells, extracellular matrix, scleraxis, cell motility, extracellular signal–regulated kinase, Ala, alanine, ECM, extracellular matrix, ERK, extracellular signal–regulated kinase, FBS, fetal bovine serum, Gly, glycine, Hyp, hydroxyproline, IPA, ingenuity pathway analysis, Leu, leucine, MAPK, mitogen-activated protein kinase, MEK, MAPK kinase, MXX, Mohawk homeobox, pAb, polyclonal antibody, PEPT1, peptide transporter 1, Pro, proline, Pro-Hyp, prolyl-4-hydroxyproline, SCX, scleraxis, SCXGFP, Scx promoter–driven GFP, SI-Pro-Hyp, stable isotopically labeled Pro-Hyp, 13C515N1-Pro-Hyp, SOX9, SRY-box transcription factor 9, TGF-β, transforming growth factor-beta, TNMD, tenomodulin

## Abstract

Collagen-derived hydroxyproline (Hyp)-containing peptides have a variety of biological effects on cells. These bioactive collagen peptides are locally generated by the degradation of endogenous collagen in response to injury. However, no comprehensive study has yet explored the functional links between Hyp-containing peptides and cellular behavior. Here, we show that the dipeptide prolyl-4-hydroxyproline (Pro-Hyp) exhibits pronounced effects on mouse tendon cells. Pro-Hyp promotes differentiation/maturation of tendon cells with modulation of lineage-specific factors and induces significant chemotactic activity *in vitro*. In addition, Pro-Hyp has profound effects on cell proliferation, with significantly upregulated extracellular signal–regulated kinase phosphorylation and extracellular matrix production and increased type I collagen network organization. Using proteomics, we have predicted molecular transport, cellular assembly and organization, and cellular movement as potential linked-network pathways that could be altered in response to Pro-Hyp. Mechanistically, cells treated with Pro-Hyp demonstrate increased directional persistence and significantly increased directed motility and migration velocity. They are accompanied by elongated lamellipodial protrusions with increased levels of active β1-integrin–containing focal contacts, as well as reorganization of thicker peripheral F-actin fibrils. Pro-Hyp–mediated chemotactic activity is significantly reduced (*p* < 0.001) in cells treated with the mitogen-activated protein kinase kinase 1/2 inhibitor PD98059 or the α5β1-integrin antagonist ATN-161. Furthermore, ATN-161 significantly inhibits uptake of Pro-Hyp into adult tenocytes. Thus, our findings document the molecular basis of the functional benefits of the Pro-Hyp dipeptide in cellular behavior. These dynamic properties of collagen-derived Pro-Hyp dipeptide could lead the way to its application in translational medicine.

Collagen is the most abundant extracellular matrix (ECM) protein in tissue/organ stroma and significantly contributes to tissue/organ integrity (1). Collagen contains at least one domain of repeated sequences of glycine (Gly)–X–Y, where X and Y are most frequently proline (Pro) and 4-hydroxyproline (Hyp), respectively (2, 3). Collagen-derived Hyp-containing peptides show a variety of physiological activities (4, 5, 6, 7, 8, 9): prolyl-4-hydroxyproline (Pro-Hyp) and alanine (Ala)–Hyp–Gly promote cell proliferation in dermal fibroblasts, whereas Pro-Hyp, Ala–Hyp–Gly, and leucine (Leu)–Hyp–Gly enhance collagen secretion in preosteoblast cells (7, 10). Hyp–Gly promotes myogenic differentiation and myotube hypertrophy (11). Leu–Hyp–Gly shows strong angiotensin-converting enzyme inhibitory activity (12). Pro-Hyp is shown to be generated by the degradation of endogenous collagen in granulation tissue to activate cells involved in tissue reconstruction/remodeling (13). The administration of gelatin hydrolysate produces a significant increase in the mean diameter of collagen fibrils in Achilles tendon (14). However, to date, no detailed study has explored the functional role of Hyp-containing peptides in tendon cell behavior. It also remains unknown whether the cellular uptake of collagen-derived peptides in tendon cells is a carrier-mediated process.

Tendon is a dense connective tissue composed of highly organized parallel and longitudinal collagen fiber bundles (15, 16, 17). Resident tendon cells (“tenocytes”) originate from multipotent mesenchymal cells and actively produce unique and tendon-specific ECM (18). Tendon stem/progenitor cells exist in normal adult human and mouse tendons (19). We have recently demonstrated that tendon progenitor cells but not residential tenocytes play a central role in the repair following adult tendon injury (20). Scleraxis (SCX) is a basic helix–loop–helix transcription factor that has been identified as a highly specific marker for tendon and ligament cells (21). The transcription of *type I collagen* (*Col1a1* and *Col1a2*) genes is specifically and directly controlled by SCX in tenocytes and cardiac fibroblasts (22, 23, 24). The tenogenic marker Scx and the chondrogenic marker SRY-box transcription factor 9 (SOX9) are known to coordinately regulate the determination of cellular lineages during embryonic development (25). Adult tenocytes in tendon/ligaments with osteoarthritis acquire chondrogenic potential, showing downregulation of SCX and upregulation of SOX9 (20, 26). Mohawk homeobox (MKX) and tenomodulin (TNMD) are also tenogenic markers during development. MKX is a transcription factor that regulates the expression of tendon-related genes such as *Scx* and *type I collagen* (27, 28, 29). TNMD is a type II transmembrane glycoprotein that is predominantly expressed in tendons and ligaments in the late stage of tendon development (30). Nevertheless, no studies to date have identified a requirement for Hyp-containing peptides in tenogenic differentiation and tenocyte maturation.

The major ECM component in tendon tissues is type I collagen (31). We have demonstrated that there are at least two independent mechanisms underlying type I collagen fibril reorganization following adult tissue injury, a fibronectin-dependent mechanism and a transforming growth factor-beta (TGF-β)/type V collagen–dependent mechanism (32, 33). Integrins are ECM receptors composed of transmembrane αβ heterodimeric subunits that mediate the organization of ECM, focal contacts, and actin-containing cytoskeleton (34, 35). A knowledge of complex integrin-mediated “outside-in” and “inside-out” signal transduction is crucial to our understanding of how integrin is associated with extracellular ligands and/or intracellular effector molecules and consequently regulates cellular behavior (36, 37, 38).

Cell migration is a dynamic process that is important for a variety of biological processes, including embryonic development, tissue repair, immune response, and tumor invasion (39, 40). Integrin engagement has a significant impact in migrating cells (41, 42): it is associated with the extension of lamellipodial and filopodial protrusions at the leading edge, where newly polymerizing actin fibers and activated but unligated integrins are clustered (43). Growing evidence reveals that the persistence of lamellipodia is a critical factor in steering cell migration and regulating cell directionality (44). The directionality of cell migration is defined as the displacement divided by the total path length of the cell; thus, if the cell is migrating more randomly, the cell directionality decreases and vice versa (45).

It remains to be elucidated how Hyp-containing peptides alter tendon cell phenotypes *in vitro*, and the precise underlying molecular mechanisms are unknown. In the present study, we hypothesized that Hyp-containing peptides have functional roles in improving cellular homeostasis in tendon cells. We recently established adult tenocyte and tendon progenitor cell lines from mouse Achilles tendon tissues that express the *scx* promoter-driven GFP as a marker, in which the expression of *Scx* promoter–driven GFP (SCXGFP) is observed when the cells differentiate into mature tenocytes (20, 46). Here, we explore the phenotypic changes in mouse tendon cells mediated by Hyp-containing peptides.

## Results

### Hyp-containing peptides enhance cell proliferation in adult tenocytes and tendon progenitor cells

**Figure 1:**
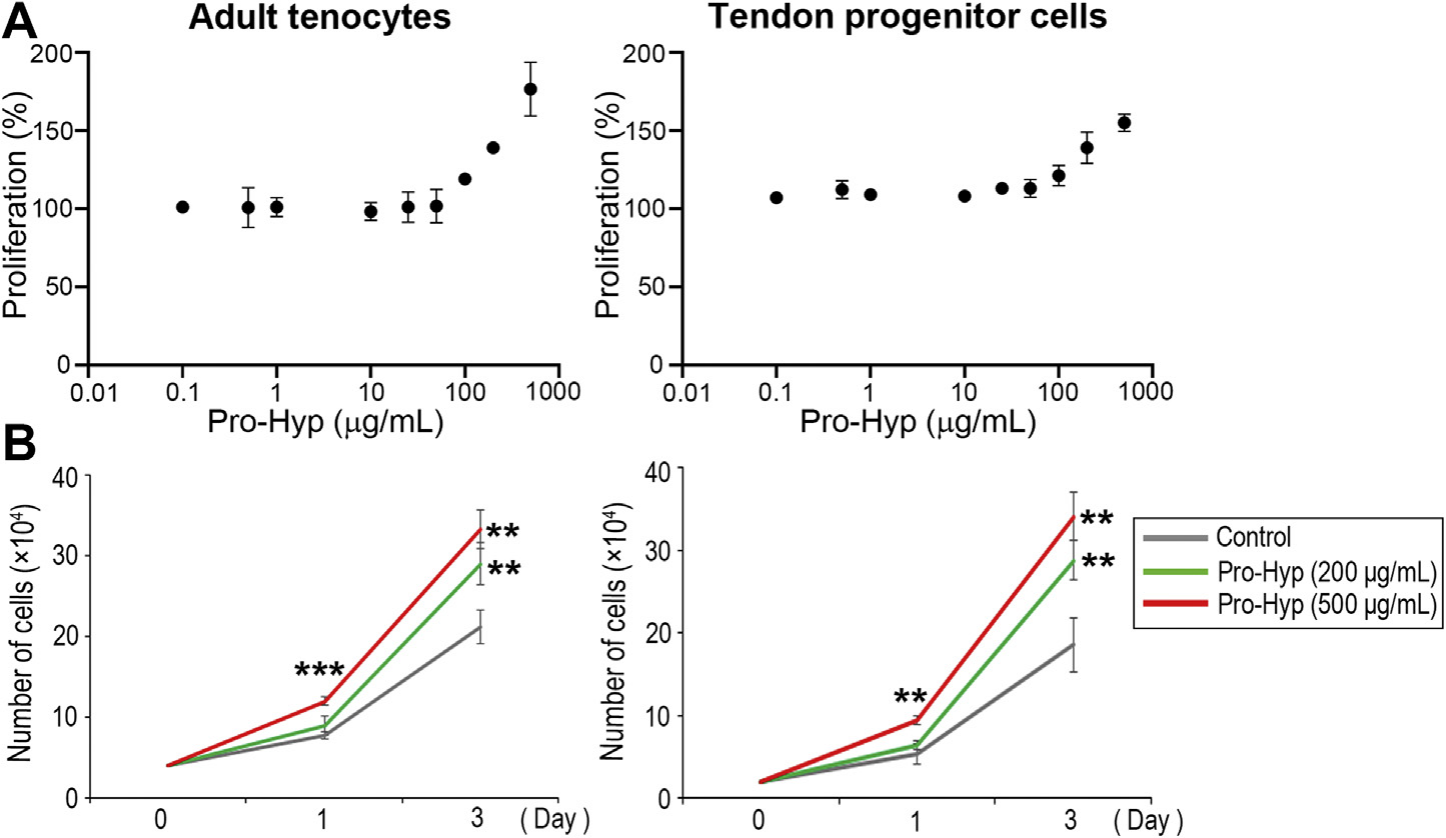
**Cell response to Pro-Hyp in adult tenocytes and tendon progenitor cells.***A*, dose responses to Pro-Hyp peptide. Cells were cultured with different concentrations of Pro-Hyp for 24 h. Dose responses are shown relative to the control value of 100% (cultured without Pro-Hyp). Error bars represent the standard deviation (n = 3). *B*, cell proliferation activity. Cells were cultured with 200 or 500 μg/ml Pro-Hyp for up to 3 days. Error bars represent the standard deviation (n = 3). ∗∗*p* < 0.01; ∗∗∗*p* < 0.001: significantly different compared with untreated controls in post hoc analysis. Pro-Hyp, prolyl-4-hydroxyproline.

The phenotypic contribution of Hyp-containing peptides to tendon cells has not yet been studied. Micro-to-millimolar concentrations of collagen-derived peptides induce chemotactic activity in a variety of cell types *in vitro* (47, 48), and food-derived collagen peptides in human peripheral blood are present in micromolar concentrations (49, 50). To study the cell response to Hyp-containing peptides, cells were initially cultured with four different Hyp-containing peptides at micromolar concentrations (Ala–Hyp–Gly, Leu–Hyp–Gly, Hyp–Gly, and Pro-Hyp; 80 μg/ml [289–462 μM]) in medium containing 5% dialyzed fetal bovine serum (FBS). Among the peptides examined, Pro-Hyp showed the greatest activity in adult tenocytes and tendon progenitor cells at day 3, which was accompanied by significant upregulation of *type I collagen* and *fibronectin* mRNA levels (Fig. S1, *A* and *B*). mRNA of the tendon-specific transcription factor *Scx* was also significantly upregulated at day 6 (Fig. S1*B*). Next, to explore the cell response to Hyp-containing peptides, the dose response to Pro-Hyp peptide was studied focusing on micromolar concentrations (0.1–500 μg/ml [0.5–2200 μM]). We found that higher concentrations, 200 and 500 μg/ml, showed more effective cell proliferation activity in both tenocytes and tendon progenitor cells (Fig. 1*A*). More specifically, Pro-Hyp significantly upregulated cell proliferation activity in both cell types in a concentration-dependent manner (Fig. 1*B*). Treatment of cells with the same concentration of l-Pro or l-Hyp did not produce any changes (data not shown). These results indicate that Pro-Hyp is the most active peptide in tendon cells. Hereafter, we focused on cellular phenotypes induced by 200 and 500 μg/ml of Pro-Hyp.

### Uptake of Pro-Hyp into adult tenocytes

**Figure 2:**
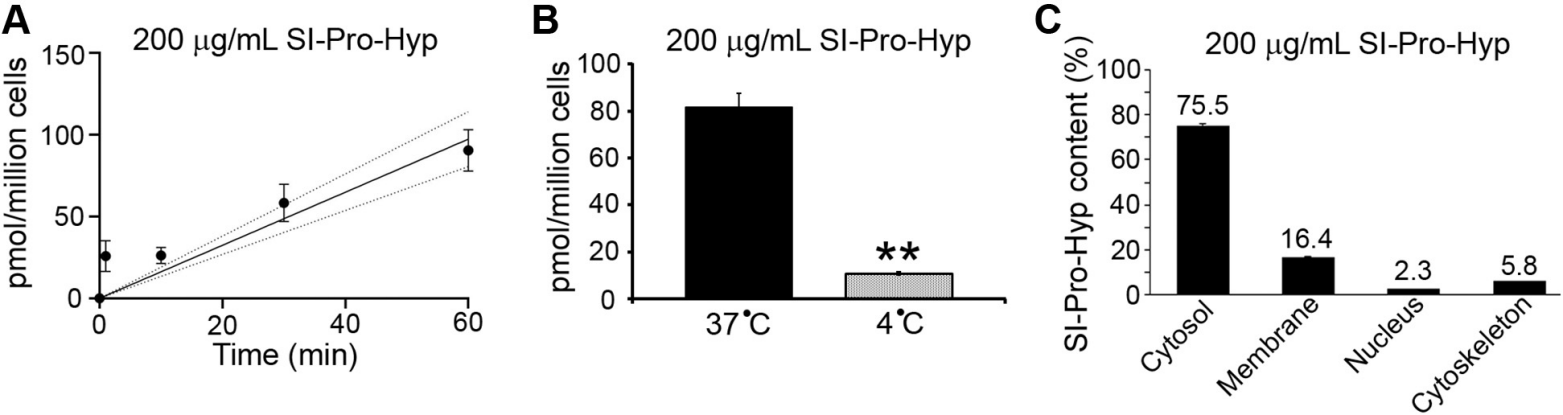
**Uptake of Pro-Hyp into adult tenocytes.***A*, time course of uptake of stable isotopically labeled Pro-Hyp (SI-Pro-Hyp; 200 μg/ml) at 37 °C in adult tenocytes. Error bars represent the standard deviation (n = 4). *B*, accumulation of SI-Pro-Hyp in tenocytes after 60 min at 37 and 4 °C. Error bars represent the standard deviation (n = 4). ∗∗*p* < 0.01. *C*, subcellular localization analysis of 200 μg/ml SI-Pro-Hyp in adult tenocytes after 60 min at 37 °C. Error bars represent the standard deviation (n = 4). Pro-Hyp, prolyl-4-hydroxyproline.

The important unsolved questions are whether Pro-Hyp is directly associated with specific receptors and activates subsequent intracellular signaling cascades, or carrier-mediated intracellular uptake of Pro-Hyp occurs, which results in the activation of inside-out signaling cascades. To study the potential for Pro-Hyp uptake into adult mouse tenocytes, a time-course assay was carried out using a stable isotopically labeled Pro-Hyp, ^13^C_5_^15^N_1_-Pro-Hyp (SI-Pro-Hyp). SI-Pro-Hyp uptake into adult mouse tenocytes exhibited transporter-mediated uptake kinetics with a linear phase of uptake (about 30 min) (Fig. 2*A*). Importantly, this uptake was significantly decreased at 4 °C (Fig. 2*B*). To further investigate this process, a kinetic analysis was performed at 37 °C in the linear phase of uptake at a fixed 30-min time point with increasing Pro-Hyp concentrations ranging from 200 to 30,000 μg/ml. However, saturation was not observed up to 30,000 μg/ml Pro-Hyp, and Pro-Hyp uptake did not follow Michaelis−Menten kinetics (data not shown). Glycylsarcosine, a typical substrate for peptide transporter 1 (PEPT1) (51), did not inhibit Pro-Hyp uptake in adult tenocytes (data not shown), indicating that the uptake of Pro-Hyp is not mediated by PEPT1.

Recently, a series of studies on cell-penetrating peptides have revealed that these peptides are incorporated into cells *via* various nonselective pathways such as endocytosis and direct translocation pathways (52, 53, 54). To study further the localization of internalized Pro-Hyp in adult tenocytes, subcellular localization analysis was carried out using SI-Pro-Hyp immediately after uptake. Interestingly, 75.5% of total SI-Pro-Hyp incorporated into cells was localized in the cytosolic fraction, 16.4% in the membranes, 5.8% in the cytoskeleton, and 2.3% in the nucleus (Fig. 2*C*) at 60 min after Pro-Hyp treatment, suggesting that tenocytes internalize Pro-Hyp through multiple pathways, including transporters and nonselective pathways.

### Pro-Hyp promotes tenogenic differentiation and maturation

**Figure 3:**
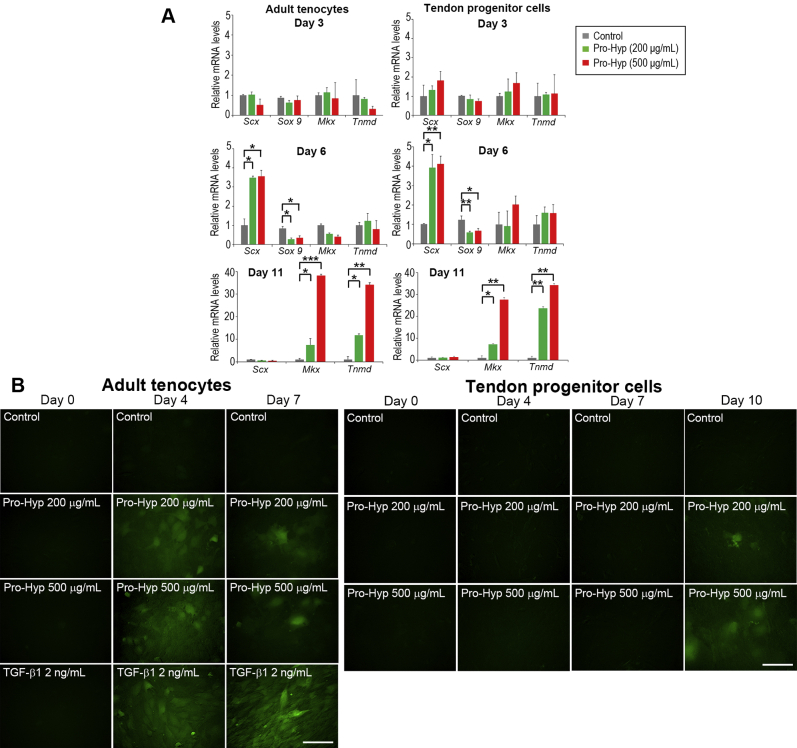
**Enhanced tenogenic differentiation in response to Pro-Hyp in adult tenocytes and tendon progenitor cells.***A*, real-time PCR analysis of S*cx*, *Sox9*, *Mkx*, and *Tnmd* mRNA levels. Cells were treated with Pro-Hyp and cultured for 3, 6, and 11 days. mRNA expression levels are shown relative to the control value of 1 (untreated cells). Error bars represent the standard deviation (n = 3). ∗*p* < 0.05; ∗∗*p* < 0.01; and ∗∗∗*p* < 0.001 (in post hoc analysis). *B*, the effect of Pro-Hyp on ScxGFP expression in adult tenocytes and tendon progenitor cells. In adult tenocyte cultures, TGF-β1 (2 ng/ml) was used as a positive control (46). The scale bars represent 100 μm. Pro-Hyp, prolyl-4-hydroxyproline; ScxGFP, *Scx* promoter–driven GFP; TGF-β1, transforming growth factor-beta.

We next explored the extent of the contribution of Pro-Hyp to differentiation and maturation in adult tenocytes and tendon progenitor cells. At day 6, mRNA of the tenogenic marker *Scx* was significantly upregulated (up to ∼4.0-fold increase compared with untreated control), whereas mRNA of the chondrogenic marker *Sox9* was significantly downregulated (∼0.3-fold compared with untreated control) in both tenocytes and tendon progenitor cells. Thereafter, *Scx* mRNA levels were markedly downregulated by day 11 (Fig. 3*A*). The mRNA levels of another tenogenic marker, *mohawk*, and of the tendon maturation marker, *tenomodulin* (55), were upregulated (up to ∼40-fold increase compared with untreated control) at a later stage (day 11) in both tenocytes and tendon progenitor cells (Fig. 3*A*). The expression levels of *type II collagen* and *aggrecan* mRNA in both cell types were undetectable even after treatment with Pro-Hyp (data not shown).

The protein expression of SCXGFP (20, 46) was further analyzed under the fluorescence microscope during the time course. From day 4 after treatment of adult tenocytes with Pro-Hyp, SCXGFP became visible, suggesting promotion to more mature tenocytes (Fig. 3*B*). Although there was only weak induction, SCXGFP had also become visible in tendon progenitor cells by day 10, confirming promotion to tendon-lineage differentiation by Pro-Hyp (Fig. 3*B*). SCXGFP was not induced in untreated cells used as negative controls (Fig. 3*B*).

### Pro-Hyp upregulates ECM production and type I collagen assembly *in vitro*

**Figure 4:**
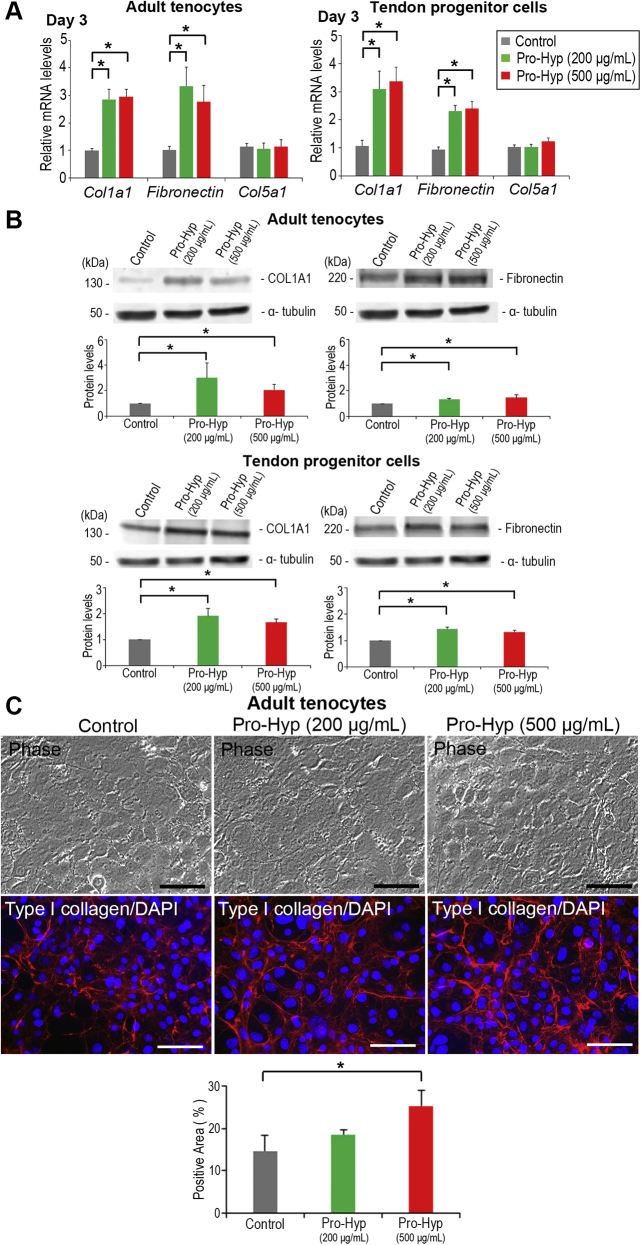
**Upregulated ECM mRNA expression and protein production and type I collagen network organization in tendon cells.***A*, real-time PCR analysis of *type I collagen* (*Col1a1*), *fibronectin*, and *type V collagen* (*Col5a1*) mRNA levels in tendon cells. Cells were cultured with Pro-Hyp for 3 days. mRNA expression levels are shown relative to the control value of 1 (untreated cells). Error bars represent the standard deviation (n = 3). ∗*p* < 0.05 (in post hoc analysis). *B*, Western blot analysis of type I collagen (COL1A1) and fibronectin in cellular lysates of tendon cells. Cells were cultured with Pro-Hyp for 3 days. Band intensity was measured by densitometry and normalized to α-tubulin (loading control). α-tubulin blots were reused in each analysis. Type I collagen and fibronectin levels are shown relative to the control value of 1 (untreated cells). Error bars represent the standard deviation (n = 3). ∗*p* < 0.05 (in post hoc analysis). *C*, enhanced type I collagen network organization in adult tenocytes. *Upper panels*, phase-contrast micrographs and immunofluorescence staining for type I collagen (*red*)/DAPI (*blue*) with Pro-Hyp for 3 days. The scale bars represent 100 μm. *Lower panel*, quantification of type I collagen positivity by densitometric analysis. Error bars represent the standard deviation (n = 3). ∗*p* < 0.05 (in post hoc analysis). DAPI, 4′,6-diamidino-2-phenylindole; ECM, extracellular matrix; Pro-Hyp, prolyl-4-hydroxyproline.

SCX is known to directly regulate the *Col1a1* and *Col1a2* genes in tendon cells (24). Since *Scx* is upregulated in response to Pro-Hyp dipeptide, the functional link between Pro-Hyp and ECM production was addressed. Adult tenocytes and tendon progenitor cells treated with Pro-Hyp for 3 days showed significantly higher mRNA expression of *type I collagen* and the type I collagen-nucleation factor *fibronectin*, whereas another nucleation factor, *type V collagen* (33, 56), was unchanged (Fig. 4*A*). The protein production of type I collagen and fibronectin was also significantly increased in cellular lysates in response to Pro-Hyp in both cell types (Fig. 4*B*), whereas type V collagen production did not show marked changes (data not shown). Pro-Hyp also affected (up to ∼1.5-fold increase) the amounts of type I collagen secreted into the culture medium (data not shown). Collagen network organization is a critical process that occurs upon tissue damage and/or remodeling (32, 57). We further explored the functional role of Pro-Hyp in the structural integrity of collagen *in vitro*. Phase-contrast image analysis showed that cells formed similar dense sheet-like structures with or without peptide treatment. Immunofluorescence analysis revealed that treatment of adult tenocytes with 500 μg/ml Pro-Hyp resulted in a significantly denser and thicker type I collagen fibril network organization compared with untreated controls (Fig. 4*C*). We have recently demonstrated that there are at least two independent mechanisms of type I collagen assembly, fibronectin mediated and type V collagen mediated (33). Thus, ∼50% increase of fibronectin production in response to 500 μg/ml Pro-Hyp shown previously may reflect the significant increase of type I collagen organization in adult tenocytes.

### Proteomics analysis and activation of extracellular signal–regulated kinase in response to Pro-Hyp

It is not yet known what the functional links are between Pro-Hyp and cell cycle progression or between cell proliferation and other signaling pathways. We next sought to understand how Pro-Hyp dipeptide modulates adult tenocyte behavior at an early time point using proteomics as a global screening tool. LC–MS/MS identified 3006 proteins across all samples (the MS proteomics data have been deposited to the ProteomeXchange Consortium *via* the PRIDE (58) partner repository with the dataset identifier PXD023176). After exposure to Pro-Hyp for 6 h, 26 proteins were significantly increased and 290 significantly decreased compared with the time-matched control sample, as revealed by ANOVA. Since the number and significance of the changes were small, the data were evaluated by functional annotation and ingenuity pathway analysis (IPA). IPA identified molecular transport, cellular assembly and organization, and cellular movement as predicted linked-network pathways at this early time point in adult tenocytes (Table S2 and Fig. S2). Therefore, these events and related signaling molecules were investigated further.

**Figure 5:**
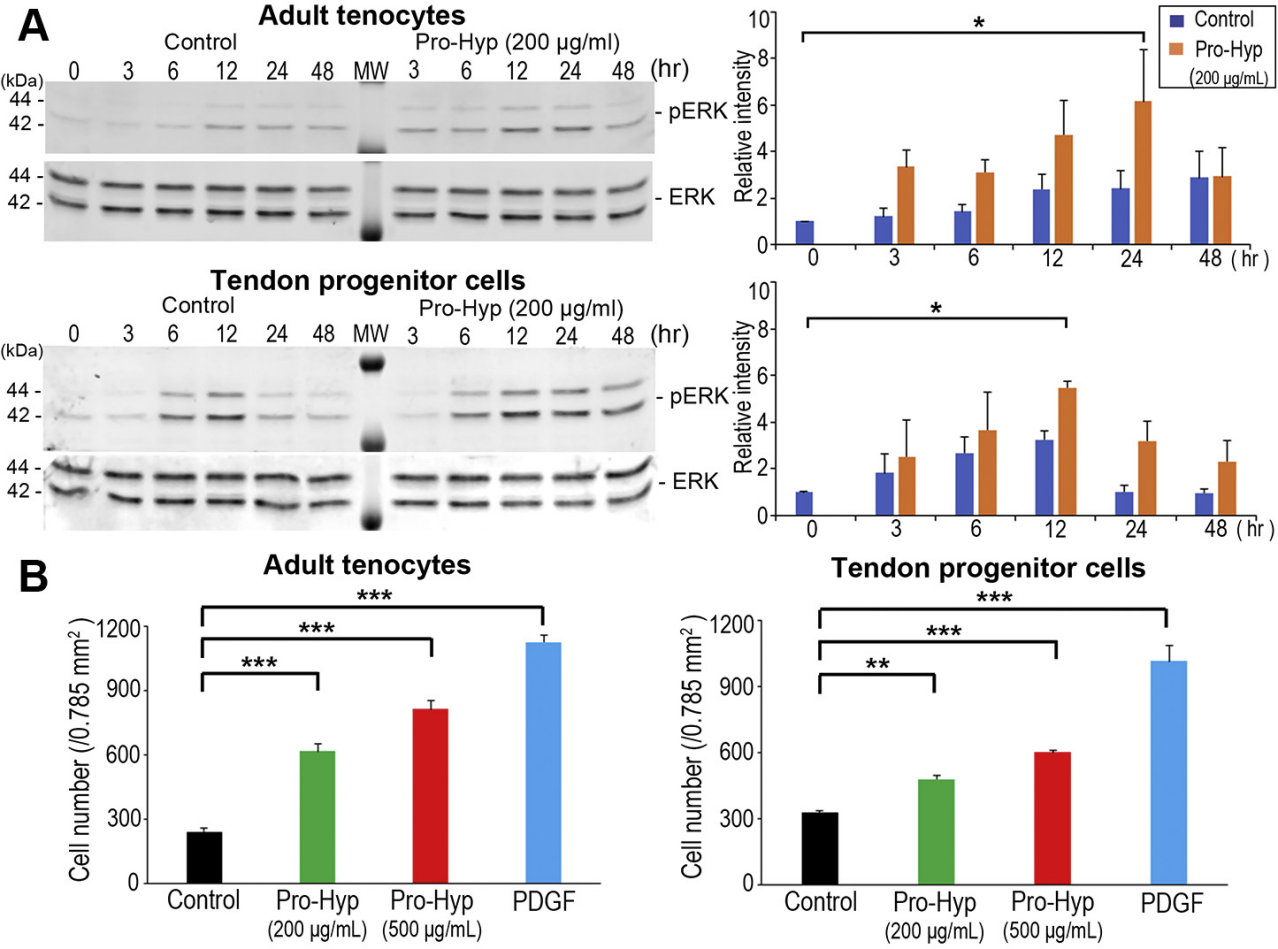
**Effect of Pro-Hyp on phospho-ERK (ERK[p202/204]) expression and cell migration activity in adult tenocytes and tendon progenitor cells.***A*, Western blot analysis of phospho-ERK and total ERK (*left panels*) and analysis of phospho-ERK intensities (*right panels*). Band intensity was measured by densitometry and normalized to total ERK. Each phospho-ERK level is shown relative to the control value of 1 (at time 0 of untreated cells). Error bars represent the standard deviation (n = 3). ∗*p* < 0.05 (in post hoc analysis). *B*, cell migration of tendon cells through gelatin-coated filters using Boyden chamber. Pro-Hyp (200 or 500 μg/ml) was added in the lower chamber. Bars represent the mean cell number/0.785 mm^2^ field. Error bars represent the standard deviation (n = 4). PDGF (10 ng/ml) was used as a positive control. ∗∗*p* < 0.01; ∗∗∗*p* < 0.001 (in post hoc analysis). ERK, extracellular signal–regulated kinase; MW, molecular weight marker; PDGF, platelet-derived growth factor; Pro-Hyp, prolyl-4-hydroxyproline.

The classical extracellular signal–regulated kinase (ERK) family (p42/44 mitogen-activated protein kinase [MAPK]; shown in Fig. S2*B*) is known to be an intracellular checkpoint for cellular mitogenesis. In cultured cells, mitogenic stimulation by growth factors correlates with stimulation of p42/44 MAPK, indicating that the ERK cascade plays a pivotal role in the control of cell cycle progression (59). Therefore, the phosphorylation level of ERK was investigated during the time course. Treatment of tendon cells with 200 μg/ml Pro-Hyp resulted in the significant upregulation of phospho-ERK: the maximum upregulation of phospho-ERK (∼6.0-fold increase) was found at 24 h after treatment in adult tenocytes and at 12 h after treatment in tendon progenitor cells, compared with untreated time 0 (Fig. 5*A*). These findings indicate the involvement of ERK signaling pathways in tendon cells treated with Pro-Hyp.

### Pro-Hyp–driven cell motility

Accumulating evidence has revealed that ERK signaling is one of the critical regulators of cell motility, although it is classically known as an important regulator of cell proliferation, differentiation, and survival through the regulation of gene expression (60). Since IPA identified cellular movement as one of the predicted linked-network pathways in response to Pro-Hyp (Fig. S2B) and Hyp-containing peptides show chemotactic activity in a variety of cell types (4, 47, 48, 61), a transwell migration assay was next performed using Boyden chambers. Adult tenocytes and tendon progenitor cells exhibited significantly enhanced chemotactic activity (up to ∼3.3-fold increase) in response to Pro-Hyp (Fig. 5*B*).

**Figure 6:**
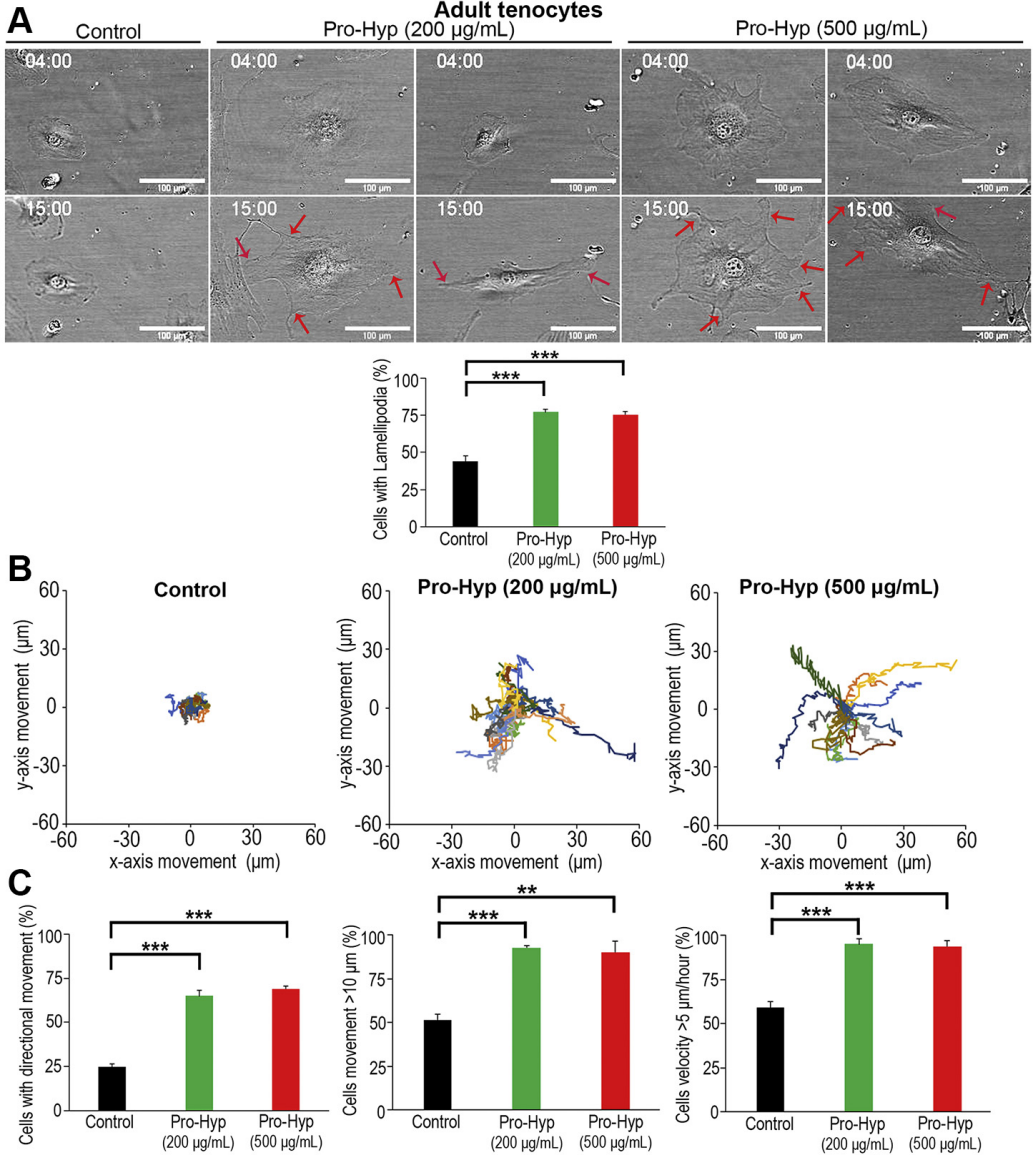
**Effect of Pro-Hyp on cell displacement and velocity in adult tenocytes.***A*, *upper panels*, time-lapse images at 4 h (the time point when Pro-Hyp was added) and 15 h in adult tenocytes. In “control,” cells were analyzed for 15-h observation periods without Pro-Hyp. *Red arrows* indicate lamellipodia-like protrusions. The scale bars represent 100 μm. *Lower panel*, percentage of cells showing lamellipodial protrusions for 11-h observation periods. Error bars represent the standard deviation. ∗∗∗*p* < 0.001 (in post hoc analysis). *B*, trajectory plots measured by cell displacement with Pro-Hyp in adult tenocytes for 11 h. The *X*- and *Y*-axes show cell movement observed every 10 min for 11 h. *C*, *left panel*, percentage of cells showing directional movement with Pro-Hyp (cells that migrate with a directionality ratio of more than 0.8; see details in Experimental procedures section). *Middle* and *right panels*, percentage of cells moving more than 10 μm (*middle panel*) and more than 5 μm/h (*right panel*) for 11-h observation periods. Error bars represent the standard deviation. ∗∗*p* < 0.01; ∗∗∗*p* < 0.001 (in post hoc analysis). Pro-Hyp, prolyl-4-hydroxyproline.

During directional migration, cells move in response to an extracellular chemotactic signal or intrinsic cues provided by the basic motility machinery (62). In order to visualize the detailed morphological changes that cells undergo during Pro-Hyp–induced migration, time-lapse image analysis of adult tenocytes was carried out. Elongated lamellipodia-like protrusions, which are broad and flat protrusions at the leading edge of cells moving on a flat substratum (44), were observed at 11 h after treatment with Pro-Hyp (Fig. 6*A* and Fig. S3). The numbers of cells with lamellipodia-like protrusions increased significantly (∼1.78-fold compared with untreated cells; Fig. 6*A*). In trajectory plots analyzing migrating tracks, cells treated with Pro-Hyp clearly demonstrated increased directional persistence compared with untreated controls (Fig. 6*B*): this resulted in a significantly increased (∼2.0-fold) number of cells with directional cell movement (cells that migrate with a directionality ratio of more than 0.8; see details in Experimental procedures section) (Fig. 6*C*). Furthermore, the numbers of cells migrating more than 10 μm and migrating more than 5 μm/h in response to Pro-Hyp were also significantly increased (Fig. 6*C*).

**Figure 7:**
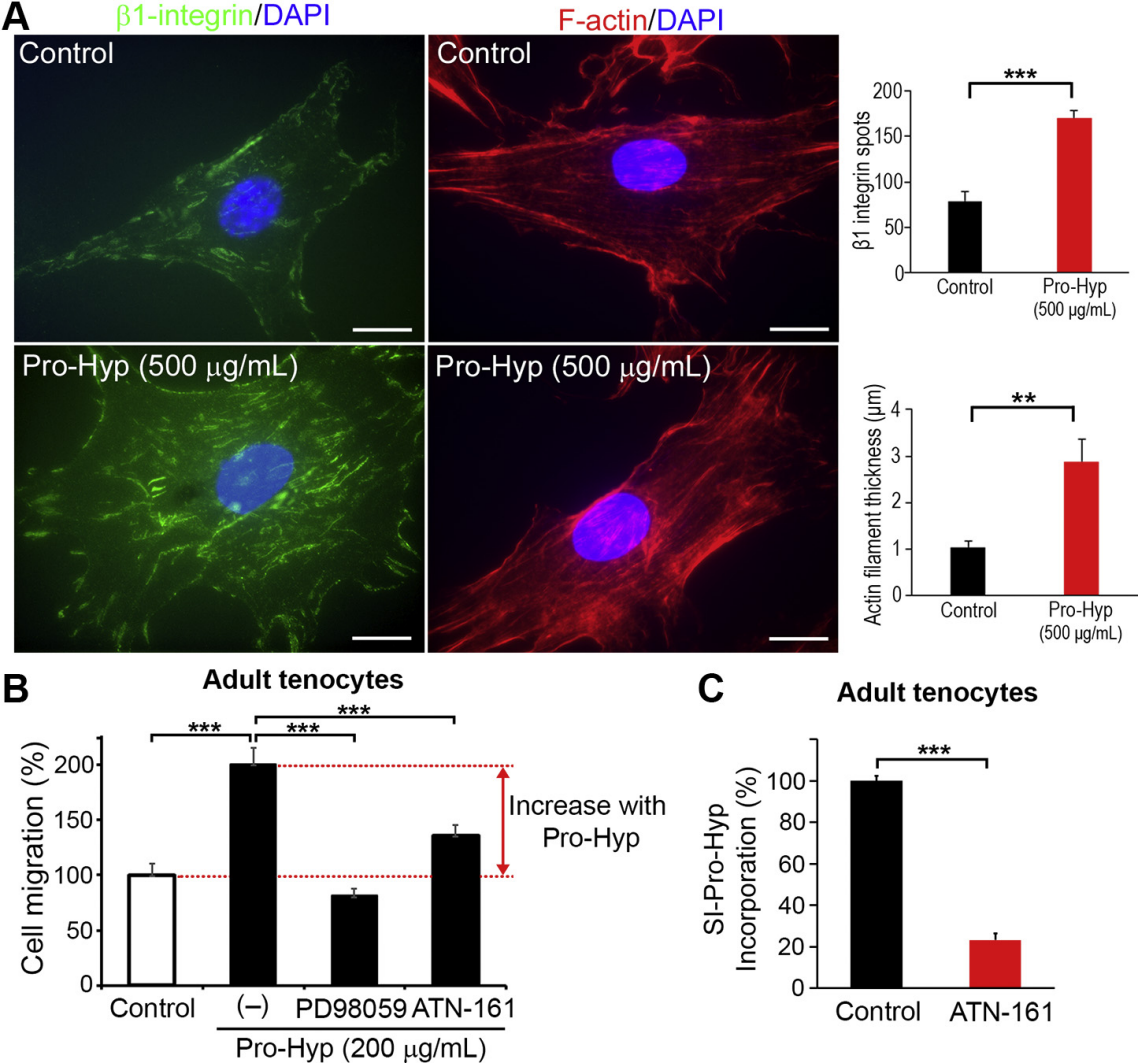
**Engagement of β1-integrin and ERK signaling in Pro-Hyp-mediated cell migration.***A*, *left panels*, immunofluorescence staining for β1-integrin (clone 9EG7, which recognizes an extracellular epitope of ligand-inducible active β1 (64): *green*)/DAPI (*blue*) and F-actin (*red*)/DAPI (*blue*). Adult tenocytes were treated with 500 μg/ml Pro-Hyp for 6 h. The scale bars represent 20 μm. *Right panels*, the numbers of active β1-integrin–containing focal contacts and the thickness of F-actin filaments when cells were treated with 500 μg/ml Pro-Hyp for 6 h. Error bars represent the standard deviation (n = 4; four separate culture experiments). ∗∗*p* < 0.01; ∗∗∗*p* < 0.001. *B*, effect of MEK1/2 inhibitor PD98059 or α5β1-integrin antagonist ATN-161 on Pro-Hyp–mediated cell migration in adult tenocytes using Boyden chamber. Cell migration activity is shown relative to the control value of 100% (cells treated with 200 μg/ml Pro-Hyp). Error bars represent the standard deviation (n = 5). ∗∗∗*p* < 0.001 (in post hoc analysis). *C*, effect of ATN-161 on uptake of stable isotopically labeled Pro-Hyp (SI-Pro-Hyp; 200 μg/ml) in adult tenocytes for 60 min at 37 °C. Error bars represent the standard deviation (n = 8). ∗∗∗*p* < 0.001. DAPI, 4′,6-diamidino-2-phenylindole; ERK, extracellular signal–regulated kinase; MEK1/2, MAPK kinase 1/2; Pro-Hyp, prolyl-4-hydroxyproline.

Since the engagement of active integrin is known to be important for protrusion formation with reorganization of the actin cytoskeleton (41, 42), the functional link between Pro-Hyp dipeptide and integrin/actin cytoskeletal organization was addressed. Integrin expression profiles in primary tenocytes from adult mouse Achilles tendon as assessed by fluorescence-activated cell sorting analysis revealed that adult tenocytes were positive for β1, α1, α5, α11, and αv integrins and very weak or negative for α6, α2, and β3 integrins (Fig. S4). There is experimental evidence that cell-matrix adhesions containing α5β1 integrin are highly dynamic, whereas adhesions containing αvβ3 are more static (63). We therefore explored the cellular distribution of the most ubiquitously expressed integrin subunit, β1, which was positive in tenocytes (using antibody 9EG7, which recognizes an extracellular epitope of ligand-inducible active β1 (64)). Immunofluorescence analysis demonstrated that treatment of adult tenocytes with 500 μg/ml Pro-Hyp resulted in a significantly increased number (∼2.2-fold) of active β1-integrin–containing focal contacts and a significantly increased thickness (∼2.8-fold) of peripheral F-actin fibrils compared with untreated controls (Fig. 7*A*).

Finally, the functional links between Pro-Hyp–induced cellular phenotypes and ERK- and β1-integrin–mediated signaling were addressed. Pretreatment of adult tenocytes with MAPK kinase (MEK)1/2 inhibitor PD98059 completely abrogated Pro-Hyp–mediated increased migratory activity (*p* < 0.001), and the magnitude of migration was decreased to a level slightly less than that of controls not treated with Pro-Hyp (Fig. 7*B*). In addition, although to a lesser degree, pretreatment of adult tenocytes with the α5β1-integrin antagonist ATN-161 (65) resulted in a reduction (∼64.2%; *p* < 0.001) in Pro-Hyp–mediated cell migration. Furthermore, ATN-161 significantly inhibited (∼76.8% inhibition) uptake of Pro-Hyp into adult tenocytes (Fig. 7*C*), suggesting Pro-Hyp as α5β1-integrin ligand and mediation of outside-in signaling. Thus, taken together, these findings indicate the involvement of ERK-mediated and β1-integrin–mediated signaling in Pro-Hyp–induced cellular phenotypes such as cell migration activity in adult tendon cells.

## Discussion

Although a previous observation suggests a functional role of specific collagen-derived peptides in the biosynthesis of matrix molecules of tendon and ligament cells (66), as of today, no molecular framework exists for understanding the mechanisms that regulate adult tendon cell phenotypes in response to those peptides. Our current study on tendon cells provides compelling evidence for the following propositions: (1) among the peptides examined, Pro-Hyp is the most effective one to induce cellular phenotypes, and the cellular uptake of Pro-Hyp is suggested to occur by multiple pathways; (2) Pro-Hyp promotes differentiation/maturation of tendon cells, enhances cell proliferation with significantly upregulated ERK-phosphorylation, and promotes type I collagen assembly *in vitro*; (3) Cells treated with Pro-Hyp demonstrate significantly increased cell motility and migration velocity, which are accompanied by elongated lamellipodial protrusions with increased active β1-integrin–containing focal contacts and thicker peripheral F-actin fibrils; and (4) Pro-Hyp–mediated chemotactic activity shows significant reduction on treatment of cells with the MEK1/2 inhibitor PD98059 or the α5β1-integrin antagonist ATN-161. Furthermore, ATN-161 significantly inhibits uptake of Pro-Hyp into adult tenocytes.

### Possible mechanisms of the cellular uptake of Pro-Hyp in tendon cells

A growing body of evidence indicates novel and unique roles of transporters in regulating metabolites and signaling molecules and in communication between organs and organisms (67). Little insight into mechanisms of the cellular uptake of Hyp-containing peptides has emerged from pharmacokinetic studies in tendon cells. In the present study, the intracellular uptake of Pro-Hyp in tendon cells is suggested to be a carrier-mediated process. Pro-Hyp–specific transporters have not yet been identified, but several candidate molecules have been suggested. One example is PEPT1, which can transport dipeptides and tripeptides (51). However, we found that glycylsarcosine, a typical substrate for PEPT1, did not inhibit Pro-Hyp uptake in adult tenocytes. Another example, the peptide/histidine transporter 1, mediates dipeptide and tripeptide uptake, and coadministration of histidine partially inhibits Hyp-Gly–induced cellular differentiation (11). Our subcellular localization analysis showed that Pro-Hyp peptide was incorporated into not only the cytosol but also the membranes/organelles, cytoskeleton, and nucleus, and α5β1-integrin antagonist ATN-161 significantly inhibited uptake of Pro-Hyp. These findings strongly suggest the existence of more than one mechanism for the intracellular incorporation of Pro-Hyp, that is, not only selective uptake mediated by transporters but also nonselective uptake mediated by endocytosis (52, 53, 54). The detailed mechanisms underlying the uptake of Hyp-containing peptides remain to be elucidated in tendon cells.

### Pro-Hyp/phospho-ERK/scx and Pro-Hyp/phospho-ERK/integrin axes in tendon cells

Collagen-derived Hyp-containing peptides can promote osteogenesis and chondrogenesis *in vitro* (8, 9, 68). Recent observations show that both fibroblast growth factor/MAPK–ERK and TGF-β/Smad2/3 signaling pathways are necessary and sufficient for *Scx* expression in chick undifferentiated limb cells, whereas inhibition of the MAPK–ERK signaling pathway is sufficient to activate *scx* in developing mouse limb progenitors (69, 70). SCX directly regulates the transcription of *type I collagen* (*Col1a1* and *Col1a2*) genes in tendon cells (24) and has been shown to be necessary and sufficient for *Tnmd* expression (21, 30, 71). This experimental evidence supports our current findings demonstrating that Pro-Hyp significantly upregulates *Scx*, *Mkx*, and *Tnmd* expression and promotes tenogenic differentiation/maturation, which results in a significant increase in type I collagen production and network organizations brought about by tendon cells. In contrast, *Scx*-null progenitors display higher chondrogenic potential with upregulation of *Sox9* (20). These findings document the counteracting effects of lineage-specific transcription factors on cellular differentiation/maturation by Pro-Hyp.

There is evidence that, when integrins are activated by ligands, endocytosis pathways through the recycling endosome are activated (72, 73). Our present findings have demonstrated ∼64.2% reduction in Pro-Hyp–mediated tenocyte migration by ATN-161. Furthermore, ATN-161 has significantly inhibited uptake of Pro-Hyp into adult tenocytes, although not complete inhibition (∼76.8%). These findings support a scenario that Pro-Hyp could mainly function as a ligand for α5β1-integrin and activate downstream ERK cascades by outside-in signaling.

Although an earlier study indicates that Pro-Hyp can abolish the suppression of the growth of skin fibroblasts on collagen gel rather than acting as a growth factor (4), the underlying molecular mechanisms have not been addressed. We have shown here the links between cell proliferation and phospho-ERK upregulation in tendon cells treated with Pro-Hyp. Recent lines of evidence have revealed the engagement of integrin-mediated inside-out signaling axes in cellular behaviors, including mediation by the MEK/ERK signaling pathway (74, 75, 76). The signaling axis by which intracellularly transported Pro-Hyp mediates enhanced cell proliferation through upregulated phospho-ERK remains to be elucidated.

### Upregulation of Pro-Hyp–driven cell migration activity in tendon cells

Although the recent observation of treating cells with mitomycin C suggests that Pro-Hyp enhances the growth of skin fibroblasts but does not enhance their motility (4), it is not clear whether Pro-Hyp can alter cell motility with the engagement of active integrins and actin reorganization in tendon cells. Integrins affect a multitude of signal transduction cascades in the control of cell proliferation and differentiation, migration, and the production of ECM (34, 36). Cell migration requires membrane protrusion at the cell front, and integrins mediate actin polymerization and organization at the leading edge of migrating cells (77). Our finding that the numbers of cells with lamellipodial protrusions were significantly increased in the presence of Pro-Hyp is consistent with the fact that lamellipodial protrusion is powered by actin polymerization, which in turn is mediated by the nucleation of branched actin networks induced by actin-related protein 2/3 (44) and by the small GTPases Cdc42 and Rac (78). The next question will be which cascades are triggered as the driving machinery to promote cell migration and actin reorganization in response to Pro-Hyp (79).

### Hyp-containing peptides for translational medicine

Adult tendon injury is a difficult clinical problem. It occurs frequently, and injured tendon heals very slowly and is rarely restored to its normal undamaged state (80, 81, 82). Tendon disorders are thus highly debilitating and painful (80). Growing evidence demonstrates that, in response to injury, Pro-Hyp is locally generated by the degradation of endogenous collagen at the wound site to activate cells involved in tissue reconstruction/remodeling ((83, 84); reviewed in Ref. (13)). Collagen-derived hydrolysates can promote the production of type III and type I collagen and proteoglycan in tendon/ligament cells *in vitro* and increase the average collagen fibril diameter in both normal and injured tendons *in vivo* (14, 66, 85). Pro-Hyp is identified as a major constituent of food-derived collagen in human blood after oral ingestion of gelatin hydrolysate (49, 50). Clinically, oral supplementation of specific collagen peptides combined with calf-strengthening exercises enhances function and reduces pain in patients with Achilles tendinopathy (86). Our current findings demonstrate several functional benefits of Pro-Hyp dipeptide at rather high concentrations (200–500 μg/ml [880–2200 μM]) in tendon cell behavior without any cytotoxic effects. These findings, taken together, make it tempting to speculate that local injections of Hyp-containing peptides could be suitable therapeutic candidates for improving the slow-healing response to adult tendon injury by promoting tendon cell differentiation/maturation and/or enhancing collagen fibrillogenesis following injury. Detailed analysis of the dynamic properties of collagen-derived peptides could shed the light on their potential application to translational medicine.

## Experimental procedures

### Cell culture

Adult mouse tendon progenitor cells and adult mouse tenocyte lines were established from primary cell cultures of Achilles tendons from 10-to-12-week-old *Scx(flox/flox)/ScxGFP/p53(−/−)* and *Scx(flox/flox)/ScxGFP/p21(−/−)* mice, as described previously (20, 46). Tendon progenitors and tenocytes were maintained in minimum essential medium Eagle alpha modification medium and Dulbecco's modified Eagle's medium, respectively, supplemented with 10% FBS and 1% nonessential amino acids. All Hyp-containing peptide experiments were performed using culture medium containing 5% dialyzed FBS. In studies performed over 3 or more days, cultures were redosed with peptides daily. Primary tenocyte cultures from adult mouse Achilles tendons were performed as described elsewhere (46).

### Reagents and antibodies

Pro-Hyp and Hyp-Gly peptides were purchased from Bachem, and other peptides were custom synthesized by AnyGen. The following antibodies were used: rabbit polyclonal antibody (pAb) against mouse type I collagen (Chemicon: this antibody shows less than 0.1% reactivity with mouse collagen types II and IV in addition to 1.0% reactivity with mouse collagen type III); rabbit pAb against mouse fibronectin (Chemicon: this antibody has crossreactivity to bovine fibronectin and shows less than 0.1% reactivity with mouse laminin and collagen types I, III, and IV by radioimmunoassay); rabbit pAb against the C-telopeptide of the α1 chain of type I collagen (LF68; provided by Dr Larry Fisher, the National Institutes of Health, USA); rabbit pAb (Abcam) and goat pAb (Thermo Fisher) against human type V collagen; mouse mAb against phospho-ERK1/2 (pT202/pY204) and rabbit pAb against total Erk (Cell Signaling); rat mAb against mouse integrin β1 (clone 9EG7) (64), hamster mAbs against mouse integrin β1 (clone Ha2/5), mouse integrin α1 (clone Ha31/8), and mouse integrin α2 (clone Ha1/29); rat mAbs against mouse integrin α5 (clone 5H10-27), mouse integrin α6 (clone GoH3), and mouse integrin αv (clone RMV-7) and hamster mAb against mouse integrin β3 (clone 2C9.G2) (all from Pharmingen); α11 (rabbit pAb; provided by Dr Donald Gullberg, University of Bergen, Norway); rabbit pAb against human calnexin (sc-11397; Santa Cruz); rabbit pAb against human Histone H1.0 (GeneTex); mouse mAb against human vimentin (clone VIM-13.2; Sigma); mouse mAb against α-tubulin (clone B-5-1-2; Sigma); and mouse mAb against rabbit GAPDH (clone 3H12; MBL). Peroxidase-conjugated donkey antimouse and anti-rabbit IgG were from Jackson ImmunoResearch Laboratories, Inc. Alexa Fluor 488 goat antirat IgG, Alexa Fluor 568 goat anti-rabbit IgG, and Alexa Fluor 568 Phalloidin were from Invitrogen. Platelet-derived growth factor from porcine platelets and human recombinant TGF-β1 were from R&D Systems. MEK1/2 inhibitor PD98059 (IC_50_ = 2 μM) was from Calbiochem, and α5β1 integrin antagonist ATN-161 was from Tocris.

### Uptake and subcellular localization analysis of Pro-Hyp in adult mouse tendon cells

Adult tenocytes were seeded at 3 × 10^5^ cells/well in 6-well plates and cultured for 12 h in growth medium. The growth medium was then replaced with transport buffer consisting of Hanks balanced salt solution, 25 mM Hepes, and 0.1% (w/v) bovine serum albumin at pH 7.4 with ^13^C_5_^15^N_1_-Pro-Hyp (SI-Pro-Hyp; 200 μg/ml) or Pro-Hyp (200–30,000 μg/ml). After incubation at 37 °C for various periods, cells were washed three times with ice-cold PBS, and cell lysates were harvested in 0.1% Triton X-100 containing protease inhibitor cocktail. After one freeze–thaw cycle and freeze-drying of the solution, the sample was reconstituted with 0.1% formic acid, and a previously developed internal standard mixture containing ^13^C_5_^15^N_1_-Pro–^13^C_5_^15^N_1_-Hyp (2SI-Pro-Hyp) was added as an internal standard (87). Then, the sample was subjected to LC–MS analysis in multipole reaction monitoring mode for quantification of SI-Pro-Hyp (*m/z* 235.2 → 75.1) and Pro-Hyp (*m/z* 229.2 → 70.1), whose concentrations were determined by the peak area ratio relative to 2SI-Pro-Hyp (*m/z* 241.2 → 75.1) as described previously (87).

To study the effect of the α5β1-integrin antagonist ATN-161 on uptake of SI-Pro-Hyp in adult tenocytes, cells were preincubated with ATN-161 (100 μM; (88)) for 60 min. Then the uptake assay was performed using 200 μg/ml SI-Pro-Hyp at 37 °C for 60 min as described previously. We confirmed that the concentration of ATN-161 used did not affect adult tenocyte proliferation (data not shown).

In subcellular localization analysis, adult tenocytes were seeded at 3 × 10^5^ cells/well in 6-well plates and cultured for 12 h in growth medium. The growth medium was then replaced with transport buffer containing 200 μg/ml of SI-Pro-Hyp. After incubation at 37 °C for 60 min, cells were washed three times with ice-cold PBS. Cells were sequentially extracted by the Calbiochem ProteoExtract Subcellular Proteome Extraction Kit (Millipore) according to the manufacturer's instructions. The accuracy of fractionation was confirmed by Western blot analysis using antibodies against GAPDH (MBL; cytosolic), calnexin (Santa Cruz; membrane/organelle), histone-1 (GeneTex; nuclear), and vimentin (Sigma; cytoskeletal) (Fig. S5). After addition of the internal standard mixture containing 2SI-Pro-Hyp, the extracted samples were diluted with 0.1% formic acid and subjected to LC–MS analysis.

### Immunofluorescence

Immunofluorescence studies were performed as described previously (89). In type I collagen network and active β1-integrin and F-actin staining, images were captured with the same gain, offset, magnitude, and exposure time. For quantification of type I collagen network–positive areas, a minimum of four different cell images were randomly selected, and their intensities were quantified using ImageJ software (version 1.48; the National Institutes of Health) (56).

### Real-time PCR

Real-time PCR was performed as described elsewhere (46, 90). The following primers were used: *Scx* forward, 5′-GAGACGGCGGCGAGAAC-3′; *Scx* reverse, 5′-TTGCTCAACTTTCTCTGGTTGCT-3′; *Sox9* forward, 5′-CGGCTCCAGCAAGAACAAG-3′; *Sox9* reverse, 5′-TGCGCCCACACCATGA-3′; *Mkx* forward, 5′-GCAGAATGGAGGGAAGGTAAG-3′; Mkx reverse, 5′-GGTTGTCACGGTGCTTGTA-3′; *Tnmd* forward, 5′-GAAACCATGGCAAAGAATCCTCCAGAG-3′; *Tnmd* reverse, 5′-TTAGACTCTCCCAAGCATGCGGGC-3′; *collagen type I* (*Col1a1*) forward, 5′-TTTGTGGACCTCCGGCTC-3′; *collagen type I* (*Col1a1*) reverse, 5′-AAGCAGAGCACTCGCCCT-3′; *collagen type V* (*Col5a1*) forward, 5′-AGGACCACACAGGGAAGC-3′; *collagen type V* (*Col5a1*) reverse, 5′-CTTGTAGACACTGAGAGCAATTCG-3′; *fibronectin* forward, 5′-GGCTCCAGATCCATCCAACAC-3′; *fibronectin* reverse, 5′-GACAGCCACTTTCACAGACAG-3′; *collagen type II* (*Col2a1*) forward, 5′-AGAACAGCATCGCCTACCTG-3′; *collagen type II* (*Col2a1*) reverse, 5′-CTTGCCCCACTTACCAGTGT-3′; *aggrecan* forward, 5′-GAGGAGAGAACTGGAGAAG-3′; *aggrecan* reverse, 5′-GGCGATAGTGGAATACAA-3′; 18S rRNA forward, 5′-GGCGACGACCCATTCG-3′; and 18S rRNA reverse, 5′-ACCCGTGGTCACCATGGTA-3′. All samples were analyzed at least in triplicate. After the reactions, the specificity of amplification in each sample was confirmed by dissociation analysis, showing that each sample gave a single melting peak. mRNA levels were normalized to the level of 18S rRNA.

### Western blot analysis

Western blot analyses were performed as described elsewhere (33). In some immunoblot analyses, samples were transferred onto an Immobilon-FL polyvinylidene fluoride membrane (Millipore Corporation) and probed with primary and IRDye 800CW– or IRDye 680–conjugated secondary antibodies (LI-COR Biosciences). Immunoreactive bands were detected using the Odyssey IR Imaging System (LI-COR Biosciences).

### Proteomics analysis

Adult tenocytes were treated with or without 500 μg/ml of Pro-Hyp for certain periods, then washed, scraped, and pelleted in phosphate buffer (pH 7.4). Subsequently, cells were resuspended in a volume of 0.5 M tetraethylammonium bromide/0.1% SDS equivalent to the volume of the cell pellet, subjected to one freeze–thaw cycle, sonicated, and centrifuged. Samples were prepared in duplicate and randomized across three isobaric tags for relative and absolute quantification (iTRAQ) experiments, with a pool of all the samples acting as a common denominator in each of the iTRAQ runs. Samples containing 100 μg protein were denatured, reduced, and treated with methyl methanethiosulfonate according to the manufacturer's protocol (Sciex), before being labeled with 8-plex iTRAQ, prefractionated by cation exchange chromatography, and analyzed on a TripleTOF 6600 Mass Spectrometer (Sciex) as previously described (91). Briefly, 40 desalted fractions were reconstituted in 0.1% formic acid, and 5 μl of each was loaded onto the column. Peptides were separated by in-line reversed phase chromatography on an Eksigent nanoLC 415 (nanoACQUITY UPLC Symmetry C18 Trap Column and an ACQUITY UPLC Peptide BEH C18 nanoACQUITY Column; Waters). A gradient from 2 to 50% acetonitrile/0.1% formic acid (v/v) over 90 min at a flow rate of 300 nl/min was applied. Spectra were acquired automatically in positive ion mode using information-dependent acquisition (Analyst TF 1.7.1. software; Sciex). Up to 25 MS/MS spectra were acquired per cycle (approximately 10 Hz) using a threshold of 500 counts per second and with dynamic exclusion for 20 s. The rolling collision energy was increased automatically by selecting the iTRAQ check box in Analyst and manually by increasing the collision energy intercepts by five. Data were searched using ProteinPilot 5.0 (Sciex) and the Paragon algorithm (Sciex) against the SwissProt database (May 2019; 17,013 mouse entries) with methylthio as a fixed modification of cysteine residues and biological modifications allowed. The mass tolerance for precursor and fragment ions was 10 ppm. The data were also searched against a reversed decoy database, and proteins lying within a 1% global false discovery rate were included in further analyses. Data from the three iTRAQ experiments were merged using RStudio V.1.0.143 (RStudio, PBC), and only proteins for which there were values in all samples were taken forward. ANOVA was performed on the natural log-transformed data using Partek Genomics Suite 7.18 (Partek Inc), with iTRAQ experiment and treatment as variables. Functional annotation cluster analysis was performed on altered proteins using the Database for Annotation, Visualization, and Integrated Discovery, with the full list of proteins identified in the combined iTRAQ data as the background list (92, 93). In addition, functional pathway prediction activity, upstream regulator analysis, and network analysis was performed on the same list of genes using the IPA software. Functional pathway prediction activity is designed to infer which functions may be activated or repressed. IPA is able to infer a functional response by comparing the observed change in protein expression with prior knowledge of expected effects between regulatory and effector genes stored in the Ingenuity Knowledge database. We applied this approach to identify which biological functions were likely to be activated or repressed as well as to infer which proteins that may not have been detected by the proteomics analysis may be responsible for driving the observed differences in the proteomic profile (upstream regulator analysis). Network analysis instead identified protein interactions that link the list of input proteins.

### Cell migration

Cell migration assays were performed in modified Boyden chambers containing Nucleopore polycarbonate membranes (5-μm pore size; Costar Corporation) as described previously (64, 94). Briefly, the filters were soaked overnight in a 100 μg/ml solution of gelatin. Chemoattractants in culture medium were added to the lower compartment of the chambers. Cells suspended in culture media were introduced into the upper compartment of the chamber. The chambers were then incubated for 6 h at 37 °C. The filters were fixed and stained, and the cells that had migrated to the lower surface were counted.

To study the effect of the MEK1/2 inhibitor PD98059 (IC_50_ = 2 μM) and the α5β1-integrin antagonist ATN-161 on Pro-Hyp–mediated cell migration in adult tenocytes, cells were preincubated for 1 h and treated with PD98059 (20 μM) or ATN-161 (100 μM; (88)). Then migration assays were performed as described previously. We confirmed that the concentration of the inhibitors used did not affect adult tenocyte proliferation (data not shown).

### Time-lapse microscopic analysis

Adult tenocytes (20,000 cells) were plated on one compartment of 3.5-cm four-compartment dishes. Cells were initially cultured for the first 4 h without peptides, then treated with 200 or 500 μg/ml of Pro-Hyp, and cultured for a further 11 h. During time-lapse assays, cells were imaged every 10 min for 15 h on a Zeiss LSM 710 confocal microscope with incubation conditions set to 37 °C and 5% CO_2_. The time-lapse settings were under transmitted light, with a 10× numerical aperture 0.45 objective, 1% laser intensity (488-nm line), 251 master gain control, and 512 × 512-pixel size. Six nonoverlapping regions of interest were randomly selected for analysis (at least 36 cells under each set of condition). We defined directional movement–positive cells as cells that migrated with a directionality ratio of more than 0.8 (displacement divided by total cell movement) (95).

### Data presentation and statistical analysis

All experiments were performed at least in triplicate on separate occasions, and the data shown were chosen as representative of results consistently observed. Results are presented as means ± SD. Differences between groups were analyzed using the two-sided Student's *t* test on raw data. In cases where more than two groups were compared, one-way ANOVA and Dunnett's post hoc test or two-way ANOVA and Tukey's post hoc test were used. A *p* value of <0.05 was considered significant. All ANOVA data are shown in Table S1.

## Data availability

All protein identifications are shown in Table S3. All remaining data are contained within the article. The MS proteomics data have been deposited to the ProteomeXchange Consortium *via* the PRIDE (https://www.ebi.ac.uk/pride/archive/) (58) partner repository with the dataset identifier PXD023176.

## Supporting information

This article contains supporting information.

## Conflict of interest

The authors declare that they have no conflicts of interest with the contents of this article.

## Supporting information

Supplemental Table S3

Supplemental Figure 3A (video)

Supplemental Figure 3B (video)

Supplemental Figure 3C (video)

Supplemental Figure 3D (video)

Supplemental Figure 3E (video)
