# Supplementary material for: The dipeptide prolyl-hydroxyproline promotes cellular homeostasis and lamellipodia-driven motility via active β1-integrin in adult tendon cells
Source: J Biol Chem. 2021 May 23;297(1):100819. doi: 10.1016/j.jbc.2021.100819 (PMC8239475; doi:10.1016/j.jbc.2021.100819)
Supplement: Supplemental Figures S1–S5 and Tables S1–S2 [file mmc1.pdf]

Supporting Information

The dipeptide prolyl-hydroxyproline promotes cellular homeostasis and lamellipodia-driven motility via active β1-integrin in adult tendon cells

Kentaro Ide, Sanai Takahashi, Keiko Sakai, Yuki Taga, Tomonori Ueno, David Dickens, Rosalind Jenkins, Francesco Falciani, Takako Sasaki, Kazuhiro Ooi, Shuichi Kawashiri, Kazunori Mizuno, Shunji Hattori, and Takao Sakai

Supplementary Figures and Legends

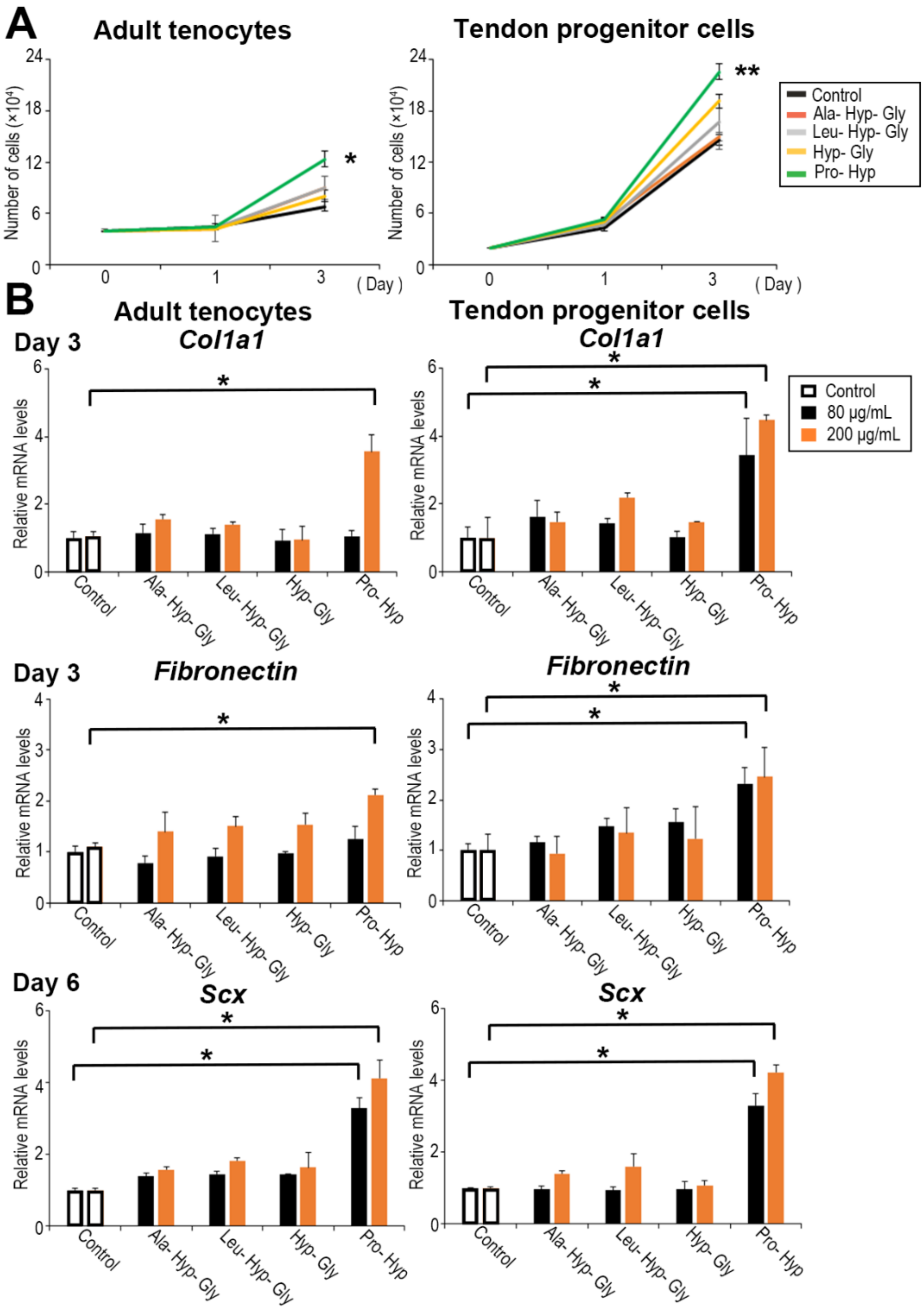

Suppl. Fig. 1. Physiological activity of Hyp-containing peptides in tendon cells.

(A) Cell proliferation assays. Adult tenocytes and tendon progenitor cells were cultured with 80 μg/mL of the Hyp-containing peptides Ala-Hyp-Gly, Leu-Hyp-Gly, Hyp-Gly, and Pro-Hyp, for up to 3 days.

(B) Real-time PCR analysis of *type I collagen* (*Col1a1)* and *fibronectin* (at day 3) and *Scx* (at day 6) mRNA levels. Cells were treated with 80 or 200 μg/mL of Hyp-containing peptides. Error bars represent the standard deviation (n = 3). *, P < 0.05; **, P < 0.01; ***, P < 0.001: significantly different compared to untreated controls (in post-hoc analysis).

Network No.	Score	Focus Molecules	Top Diseases and Functions
1	65	30	[Molecular Transport, Protein Trafficking, RNA Post-Transcriptional Modification]
2	51	25	[Cell Morphology, Organismal Injury and Abnormalities, Skeletal and Muscular Disorders]
3	45	23	[Cellular Assembly and Organization, Molecular Transport, RNA Trafficking]
4	37	20	[Cellular Movement, Embryonic Development, Renal and Urological System Development and Function]
5	26	15	[Cell Signaling, Cellular Assembly and Organization, Cellular Function and Maintenance]
6	21	13	[Cell Morphology, Cellular Assembly and Organization, DNA Replication, Recombination, and Repair]
7	21	13	[Cellular Development, Cellular Movement, Reproductive System Development and Function]
8	19	12	[Cancer, Post-Translational Modification, Protein Folding]

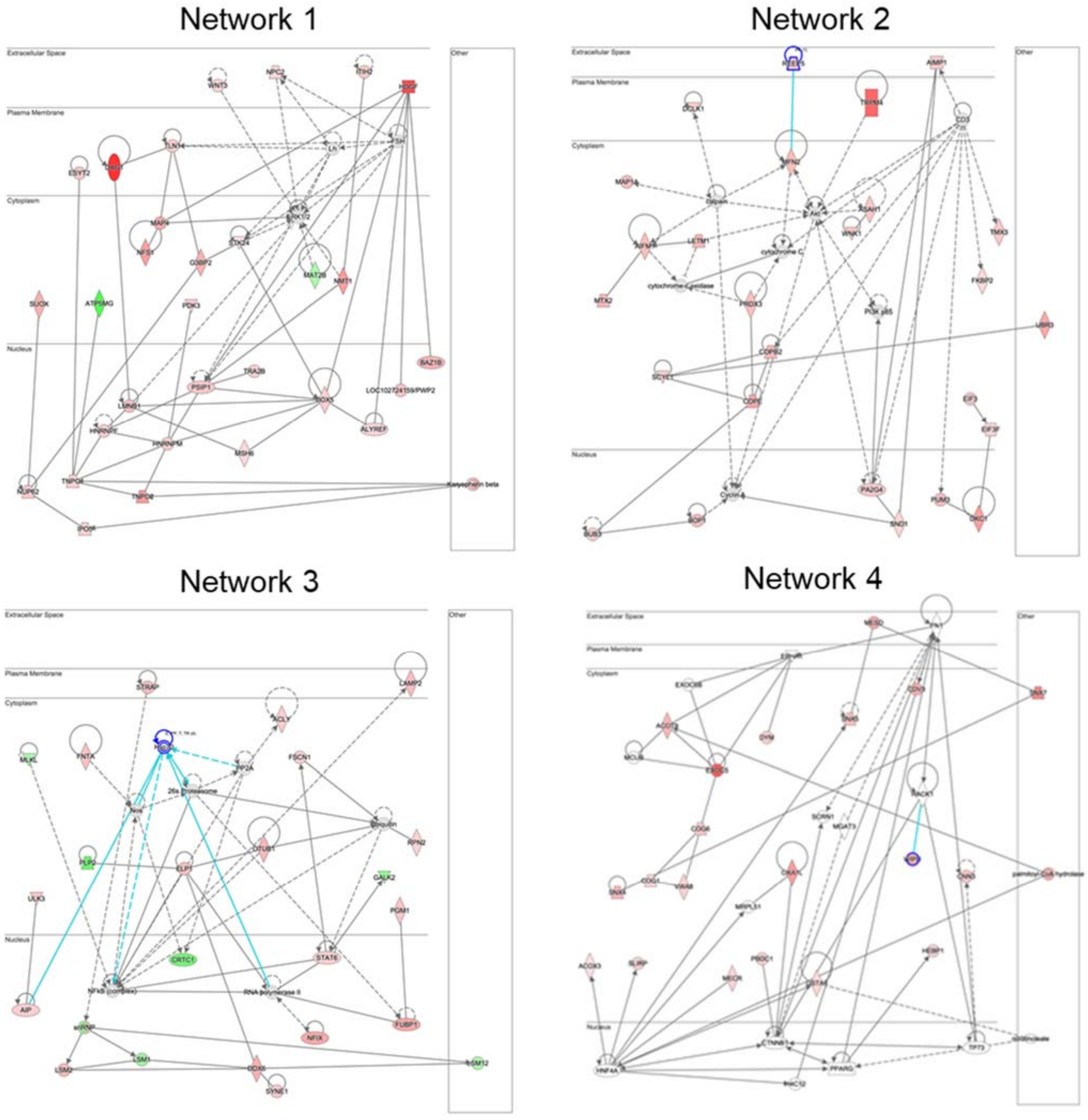

Suppl. Fig. 2. Proteomics analysis in adult tenocytes.
(**Upper panel**) The 8 networks that Ingenuity Pathway Analysis (IPA) identified linking the proteins differentially regulated at 6 hrs after Pro-Hyp treatment. Columns show the network ID, score, number of proteins included in each network and functions enriched in each network. (**Lower panels**) The 4 significant IPA networks identified using the list of proteins differentially regulated at 6 hrs after Pro-Hyp treatment. The network number represents each network described in the upper panel.

Suppl. Fig. 3. Live-cell imaging: Time-lapse microscopic analysis of adult tenocytes for 15 hrs. Adult tenocytes were (**A**) left untreated for 15 hrs or treated with (**B, C**) 200 µg/mL or (**D, E**) 500 µg/mL Pro-Hyp at the 4-hr time point and further observed for 11 hrs (total 15 hrs). The data are shown in separate video files (.avi). Scale bar, 100 µm.

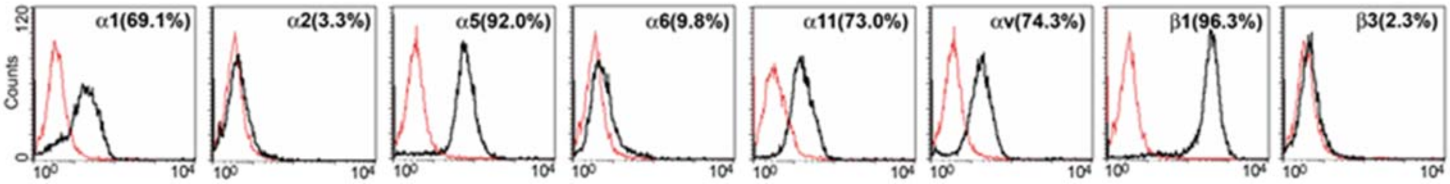

Suppl. Fig. 4. Integrin expression profiles in adult mouse primary tenocytes by FACS analysis. Adult mouse primary cultured tenocytes were stained with the anti-integrin antibodies α1 (clone Ha31/8), α2 (clone Ha1/29), α5 (clone 5H10-27), α6 (clone GoH3), α11, αv (clone RMV-7), β1 (clone Ha2/5), or β3 (clone 2C9.G2). Red and black histograms denote control fluorescence and cell/antibody-bound fluorescence, respectively. The percentage of positive cells is indicated in each panel.

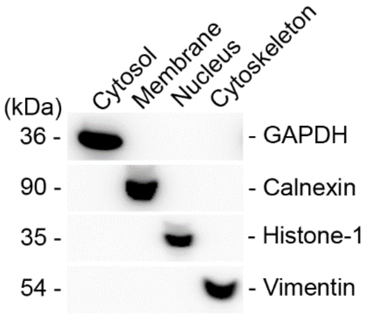

Suppl. Fig. 5. Western blot analysis of GAPDH (cytosolic marker), Calnexin (membrane), Histone-1 (nucleus), and Vimentin (cytoskeleton) after fractionation of cytosol, membrane/organelle, nucleus, and cytoskeleton from adult tenocytes.

Suppl. Table 1. Results of the ANOVA analyses

Figure	Statistics	ANOVA, F value	ANOVA, P value
**Fig. 1B**	Adult tenocytes, day 1	22.89	0.001
	Adult tenocytes, day 3	24.41	0.001
	Progenitors, day 1	20.8	0.002
	Progenitors, day 3	22.07	0.001
**Fig. 3A**	Adult tenocyts, day 6, Scx	43.02	< 0.001
	Adult tenocytes, day 6, Sox 9	13.17	0.002
	Progenitors, day 6, Scx	7.35	0.005
	Progenitors, day 6, Sox 9	6.8	0.008
	Adult tenocytes, day 11, Mkt	19.23	0.002
	Adult tenocytes, day 11, Tnmd	33.79	< 0.001
	Progenitors, day 11, Mkt	18.77	0.002
	Progenitors, day 11, Tnmd	20.95	0.001
**Fig. 4A**	Adult tenocytes, Col1a1	5.25	0.012
	Adult tenocytes, Fibronectin	4.43	0.025
	Progenitors, Col1a1	6.19	0.02
	Progenitors, Fibronectin	6.727	0.029
**Fig. 4B**	Adult tenocytes, COL1A1	5.8	0.019
	Adult tenocytes, Fibronectin	5.61	0.026
	Progenitors, COL1A1	6.81	0.007
	Progenitors, Fibronectin	15.62	0.001
**Fig. 4C**	Adult tenocytes, Col positive area	12.39	0.007
**Fig. 5A#**	Adult tenocytes, Control vs Pro-Hyp treated	8.39	0.008
	Adult tenocytes, Time course	2.62	0.05
	Adult tenocytes, Interaction	1.04	0.42
	Progenitors, Control vs Pro-Hyp treated	6.87	0.015
	Progenitors, Time course	4.26	0.007
	Progenitors, Interaction	0.56	0.73
**Fig. 5B**	Progenitors, Migration	66.42	< 0.001
**Fig. 6A**	Cells with lamellipodia	148.2	< 0.001
**Fig. 6C**	Directional movement	112.6	< 0.001
	Movement > 10 μm	13.09	< 0.001
	Velocity > 5 μm/hour	38.09	< 0.001
**Fig. 7B**	Cell migration	98.94	<0.001

**Suppl. Fig. 1A**	Adult tenocytes, day 3	5.38	0.014
	Progenitors, day 3	4.07	0.032
**Suppl. Fig. 1B**	Adult tenocytes, Col1a1 (200 μg/mL peptide)	12.67	< 0.001
	Progenitors, Col1a1 (80 μg/mL peptide)	4.05	0.033
	Progenitors, Col1A1 (200 mg/mL peptide)	8.95	0.006
	Adult tenocytes, Fibronectin (200 μg/mL peptide)	3.85	0.038
	Progenitors, Fibronectin (80 μg/mL peptide)	13.14	< 0.001
	Progenitors, Fibronectin (200 μg/mL peptide)	7	0.012
	Adult tenocytes, Scx (80 μg/mL peptide)	3.98	0.034
	Adult tenocytes, Scx (80 μg/mL peptide)	4.35	0.027
	Progenitors, Scx (80 μg/mL peptide)	4.95	0.018
	Progenitors, Scx (200 μg/mL peptide)	9.16	0.002

#, Data in Fig. 5A were analyzed using Two-way ANOVA.

Suppl. Table 2. Proteomics analysis in adult tenocytes. List of proteins where the fold change is significant for at least two time points by two-way ANOVA using the Database for Annotation, Visualization and Integrated Discovery (DAVID).

Protein	Accession	p-value	Ratio	Fold-Change	Fold-Change (Description)
Dystroglycan	Q62165	0.044	0.115	-8.681	6h + down vs 6h -
Mitochondrial import inner membrane translocase subunit Tim9	Q9WV98	0.049	0.118	-8.451	6h + down vs 6h -
ATP synthase subunit gamma, mitochondrial	Q91VR2	0.049	0.120	-8.358	6h + down vs 6h -
Hepatoma-derived growth factor	P51859	0.042	0.131	-7.647	6h + down vs 6h -
Exocyst complex component 5	Q3TPX4	0.027	0.148	-6.764	6h + down vs 6h -
RNA-binding protein Raly	Q64012	0.041	0.151	-6.641	6h + down vs 6h -
Transient receptor potential cation channel subfamily M member 4	Q7TN37	0.027	0.157	-6.360	6h + down vs 6h -
Protein virilizer homolog	A2AIV2	0.016	0.182	-5.500	6h + down vs 6h -
Myristoylated alanine-rich C-kinase substrate	P26645	0.018	0.190	-5.270	6h + down vs 6h -
Exocyst complex component 6B	A6H5Z3	0.033	0.192	-5.212	6h + down vs 6h -
Epidermal growth factor receptor kinase substrate 8-like protein 2	Q99K30	0.039	0.192	-5.198	6h + down vs 6h -
Sorting nexin-7	Q9CY18	0.026	0.196	-5.093	6h + down vs 6h -
Nucleoside diphosphate kinase B	Q01768	0.007	0.205	-4.885	6h + down vs 6h -
Cysteine and histidine-rich domain-containing protein 1	Q9D1P4	0.037	0.211	-4.739	6h + down vs 6h -
Endoribonuclease LACTB2	Q99KR3	0.035	0.213	-4.700	6h + down vs 6h -
Serine/threonine-protein kinase SIK3	Q6P4S6	0.047	0.214	-4.664	6h + down vs 6h -
NADH dehydrogenase [ubiquinone] 1 alpha subcomplex subunit 8	Q9DCJ5	0.041	0.218	-4.579	6h + down vs 6h -
60S ribosomal protein L35	Q6ZWV7	0.025	0.226	-4.427	6h + down vs 6h -
Beta-arrestin-1	Q8BWG8	0.033	0.228	-4.378	6h + down vs 6h -
Transportin-2	Q99LG2	0.003	0.233	-4.297	6h + down vs 6h -
Protein Noxp20	Q9D281	0.012	0.234	-4.278	6h + down vs 6h -
Vesicle-associated membrane protein-associated protein A	Q9WV55	0.015	0.235	-4.258	6h + down vs 6h -
Parathymosin	Q9D0J8	0.030	0.237	-4.228	6h + down vs 6h -
Glycylpeptide N-tetradecanoyltransferase 1	O70310	0.038	0.239	-4.183	6h + down vs 6h -
Cleavage and polyadenylation specificity factor subunit 3	Q9QXK7	0.042	0.241	-4.149	6h + down vs 6h -
Cohesin subunit SA-2	O35638	0.034	0.242	-4.132	6h + down vs 6h -
5'-AMP-activated protein kinase subunit beta-1	Q9R078	0.029	0.246	-4.062	6h + down vs 6h -
Mitochondrial inner membrane protein OXA1L	Q8BGA9	0.043	0.247	-4.053	6h + down vs 6h -
H/ACA ribonucleoprotein complex subunit DKC1	Q9ESX5	0.037	0.248	-4.038	6h + down vs 6h -
Serine/threonine-protein phosphatase 2A 65 kDa regulatory subunit A alpha isoform	Q76MZ3	0.033	0.249	-4.013	6h + down vs 6h -
Cysteine desulfurase, mitochondrial	Q9Z1J3	0.040	0.250	-3.997	6h + down vs 6h -
Eukaryotic translation initiation factor 2 subunit 2	Q99L45	0.012	0.257	-3.896	6h + down vs 6h -
THUMP domain-containing protein 3	P97770	0.002	0.257	-3.893	6h + down vs 6h -
Constitutive coactivator of PPAR-gamma-like protein 2	Q8C3F2	0.039	0.257	-3.889	6h + down vs 6h -
S-formylglutathione hydrolase	Q9R0P3	0.021	0.263	-3.800	6h + down vs 6h -
X-ray repair cross-complementing protein 5	P27641	0.025	0.265	-3.780	6h + down vs 6h -
Far upstream element-binding protein 1	Q91WJ8	0.027	0.270	-3.709	6h + down vs 6h -
WD repeat domain phosphoinositide-interacting protein 1	Q8R3E3	0.025	0.270	-3.699	6h + down vs 6h -
Plasminogen activator inhibitor 1 RNA-binding protein	Q9CY58	0.023	0.271	-3.687	6h + down vs 6h -
Coatomer subunit epsilon	O89079	0.026	0.273	-3.668	6h + down vs 6h -
E3 ubiquitin-protein ligase UBR3	Q5U430	0.017	0.274	-3.647	6h + down vs 6h -
Nuclear factor 1 X-type	P70257	0.019	0.276	-3.628	6h + down vs 6h -
LRP chaperone MESD	Q9ERE7	0.028	0.276	-3.623	6h + down vs 6h -
Microtubule-associated protein 4	P27546	0.017	0.278	-3.599	6h + down vs 6h -
Histone-binding protein RBBP4	Q60972	0.046	0.280	-3.572	6h + down vs 6h -
Cytoskeleton-associated protein 5	A2AGT5	0.028	0.283	-3.528	6h + down vs 6h -
Anaphase-promoting complex subunit 1	P53995	0.028	0.284	-3.527	6h + down vs 6h -
Aldose reductase-related protein 2	P45377	0.022	0.285	-3.511	6h + down vs 6h -
COMM domain-containing protein 4	Q9CQ02	0.032	0.291	-3.439	6h + down vs 6h -
Cytochrome b-c1 complex subunit 7	Q9D855	0.036	0.294	-3.400	6h + down vs 6h -
Sulfite oxidase, mitochondrial	Q8R086	0.031	0.296	-3.378	6h + down vs 6h -
Protein CDV3	Q4VAA2	0.044	0.297	-3.364	6h + down vs 6h -
Ran-binding protein 3	Q9CT10	0.029	0.302	-3.315	6h + down vs 6h -
Probable ATP-dependent RNA helicase DDX6	P54823	0.019	0.304	-3.289	6h + down vs 6h -
N-alpha-acetyltransferase 35, NatC auxiliary subunit	Q6PHQ8	0.011	0.304	-3.287	6h + down vs 6h -
Dual specificity mitogen-activated protein kinase kinase 4	P47809	0.009	0.305	-3.277	6h + down vs 6h -
Rabankyrin-5	Q810B6	0.046	0.305	-3.274	6h + down vs 6h -
Tyrosine-protein kinase BAZ1B	Q9Z277	0.035	0.307	-3.255	6h + down vs 6h -

Ras GTPase-activating protein-binding protein 2	P97379	0.022	0.309	-3.241	6h + down vs 6h -
Dedicator of cytokinesis protein 7	Q8R1A4	0.045	0.309	-3.231	6h + down vs 6h -
Mitochondrial fission 1 protein	Q9CQ92	0.038	0.319	-3.138	6h + down vs 6h -
Torsin-1B	Q9ER41	0.037	0.320	-3.129	6h + down vs 6h -
Elongation factor Ts, mitochondrial	Q9CZR8	0.013	0.321	-3.120	6h + down vs 6h -
Phosphoacetylglucosamine mutase	Q9CYR6	0.028	0.321	-3.115	6h + down vs 6h -
D-aminoacyl-tRNA deacylase 2	Q8BHA3	0.038	0.321	-3.115	6h + down vs 6h -
Elongation factor G, mitochondrial	Q8K0D5	0.046	0.323	-3.099	6h + down vs 6h -
Elongation factor 1-delta	P57776	0.014	0.323	-3.097	6h + down vs 6h -
Acyl-coenzyme A thioesterase 9, mitochondrial	Q9R0X4	0.049	0.324	-3.090	6h + down vs 6h -
Sorting nexin-4	Q91YJ2	0.025	0.324	-3.084	6h + down vs 6h -
14-3-3 protein gamma	P61982	0.028	0.324	-3.082	6h + down vs 6h -
Ribosomal L1 domain-containing protein 1	Q8BVY0	0.014	0.325	-3.075	6h + down vs 6h -
Mth938 domain-containing protein	Q8R0P4	0.002	0.327	-3.062	6h + down vs 6h -
Zinc finger C2HC domain-containing protein 1A	Q8BJH1	0.018	0.329	-3.043	6h + down vs 6h -
Coatomer subunit beta'	O55029	0.019	0.329	-3.040	6h + down vs 6h -
Ubiquitin thioesterase OTUB1	Q7TQI3	0.027	0.332	-3.010	6h + down vs 6h -
Mitochondrial proton/calcium exchanger protein	Q9Z2I0	0.039	0.334	-2.993	6h + down vs 6h -
Methyltransferase-like 26	Q9DCS2	0.034	0.335	-2.986	6h + down vs 6h -
Thyroid hormone receptor-associated protein 3	Q569Z6	0.039	0.335	-2.985	6h + down vs 6h -
Metaxin-2	O88441	0.047	0.336	-2.980	6h + down vs 6h -
Ezrin	P26040	0.050	0.338	-2.960	6h + down vs 6h -
Cytosolic acyl coenzyme A thioester hydrolase	Q91V12	0.023	0.342	-2.925	6h + down vs 6h -
Host cell factor 1	Q61191	0.042	0.343	-2.920	6h + down vs 6h -
Caprin-1	Q60865	0.010	0.348	-2.876	6h + down vs 6h -
Eukaryotic translation initiation factor 5B	Q05D44	0.032	0.352	-2.845	6h + down vs 6h -
Serine-threonine kinase receptor-associated protein	Q9Z1Z2	0.004	0.354	-2.825	6h + down vs 6h -
U6 snRNA-associated Sm-like protein LSm2	O35900	0.014	0.356	-2.809	6h + down vs 6h -
Microtubule-associated protein 1A	Q9QYR6	0.025	0.357	-2.799	6h + down vs 6h -
Pumilio homolog 3	Q8BKS9	0.009	0.361	-2.772	6h + down vs 6h -
Anaphase-promoting complex subunit 2	Q8BZQ7	0.003	0.361	-2.770	6h + down vs 6h -
Ribosome biogenesis protein BOP1	P97452	0.030	0.361	-2.770	6h + down vs 6h -
Lysosome-associated membrane glycoprotein 2	P17047	0.035	0.362	-2.762	6h + down vs 6h -
Protein transport protein Sec16A	E9QAT4	0.031	0.364	-2.750	6h + down vs 6h -
Mitofusin-2	Q80U63	0.000	0.364	-2.745	6h + down vs 6h -
Hemoglobin subunit alpha	P01942	0.033	0.365	-2.741	6h + down vs 6h -
Lamin-B1	P14733	0.021	0.366	-2.733	6h + down vs 6h -
Aconitate hydratase, mitochondrial	Q99KI0	0.037	0.366	-2.730	6h + down vs 6h -
14-3-3 protein zeta/delta	P63101	0.009	0.366	-2.730	6h + down vs 6h -
Fumarate hydratase, mitochondrial	P97807	0.041	0.370	-2.706	6h + down vs 6h -
Apoptosis-inducing factor 1, mitochondrial	Q9Z0X1	0.031	0.371	-2.693	6h + down vs 6h -
Sorting nexin-5	Q9D8U8	0.037	0.372	-2.691	6h + down vs 6h -
Thioredoxin-dependent peroxide reductase, mitochondrial	P20108	0.043	0.372	-2.688	6h + down vs 6h -
Protein farnesyltransferase/ geranylgeranyltransferase type-1 subunit alpha	Q61239	0.038	0.374	-2.673	6h + down vs 6h -
Protein Niban	Q3UW53	0.004	0.374	-2.671	6h + down vs 6h -
Eukaryotic translation initiation factor 5	P59325	0.045	0.375	-2.670	6h + down vs 6h -
Elongator complex protein 1	Q7TT37	0.025	0.379	-2.641	6h + down vs 6h -
Ataxin-10	P28658	0.026	0.380	-2.630	6h + down vs 6h -
Structural maintenance of chromosomes flexible hinge domain-containing protein 1	Q6P5D8	0.018	0.382	-2.616	6h + down vs 6h -
Probable 2-oxoglutarate dehydrogenase E1 component DHKTD1, mitochondrial	A2ATU0	0.042	0.384	-2.605	6h + down vs 6h -
Protein PRRC2C	Q3TLH4	0.024	0.385	-2.600	6h + down vs 6h -
V-type proton ATPase subunit C 1	Q9Z1G3	0.034	0.386	-2.590	6h + down vs 6h -
Nucleotide exchange factor SIL1	Q9EPK6	0.022	0.386	-2.590	6h + down vs 6h -
Serine/threonine-protein phosphatase 4 regulatory subunit 2	Q0VGB7	0.011	0.386	-2.589	6h + down vs 6h -
14-3-3 protein beta/alpha	Q9CQV8	0.009	0.387	-2.587	6h + down vs 6h -
Neurolysin, mitochondrial	Q91YP2	0.002	0.390	-2.565	6h + down vs 6h -
Farnesyl pyrophosphate synthase	Q920E5	0.018	0.393	-2.541	6h + down vs 6h -
Anaphase-promoting complex subunit 5	Q8BTZ4	0.029	0.395	-2.534	6h + down vs 6h -
Receptor expression-enhancing protein 5	Q60870	0.019	0.396	-2.527	6h + down vs 6h -
Serine/threonine-protein kinase ULK3	Q3U3Q1	0.030	0.398	-2.510	6h + down vs 6h -
LIM domain and actin-binding protein 1	Q9ERG0	0.032	0.399	-2.505	6h + down vs 6h -
Adenosine deaminase	P03958	0.004	0.399	-2.504	6h + down vs 6h -
Segment polarity protein dishevelled homolog DVL-2	Q60838	0.042	0.401	-2.494	6h + down vs 6h -
Serine/threonine-protein kinase WNK1	P83741	0.013	0.402	-2.486	6h + down vs 6h -
Periodic tryptophan protein 2 homolog	Q8BU03	0.039	0.404	-2.473	6h + down vs 6h -

Conserved oligomeric Golgi complex subunit 6	Q8R3I3	0.030	0.405	-2.468	6h + down vs 6h -
Protein arginine N-methyltransferase 1	Q9JIF0	0.028	0.405	-2.467	6h + down vs 6h -
Apoptotic chromatin condensation inducer in the nucleus	Q9JIX8	0.024	0.408	-2.451	6h + down vs 6h -
Calponin-3	Q9DAW9	0.039	0.409	-2.444	6h + down vs 6h -
Catenin alpha-1	P26231	0.023	0.410	-2.438	6h + down vs 6h -
NAD(P) transhydrogenase, mitochondrial	Q61941	0.045	0.411	-2.435	6h + down vs 6h -
Beta-1,3-glucosyltransferase	Q8BHT6	0.047	0.411	-2.432	6h + down vs 6h -
Nuclear pore complex protein Nup155	Q99P88	0.033	0.412	-2.428	6h + down vs 6h -
Extended synaptotagmin-2	Q3TZZ7	0.038	0.416	-2.405	6h + down vs 6h -
Methylmalonyl-CoA mutase, mitochondrial	P16332	0.012	0.418	-2.393	6h + down vs 6h -
Phosphoglucomutase-1	Q9D0F9	0.016	0.418	-2.392	6h + down vs 6h -
40S ribosomal protein S27	Q6ZWU9	0.018	0.418	-2.392	6h + down vs 6h -
Basigin	P18572	0.016	0.422	-2.369	6h + down vs 6h -
COP9 signalosome complex subunit 5	O35864	0.044	0.422	-2.368	6h + down vs 6h -
Pleckstrin homology-like domain family B member 2	Q8K1N2	0.015	0.423	-2.365	6h + down vs 6h -
Interleukin enhancer-binding factor 2	Q9CXY6	0.021	0.423	-2.364	6h + down vs 6h -
Proliferation-associated protein 2G4	P50580	0.035	0.425	-2.352	6h + down vs 6h -
Acid ceramidase	Q9WV54	0.041	0.425	-2.351	6h + down vs 6h -
Trifunctional purine biosynthetic protein adenosine-3	Q64737	0.040	0.427	-2.342	6h + down vs 6h -
Septin-11	Q8C1B7	0.025	0.428	-2.334	6h + down vs 6h -
PC4 and SFRS1-interacting protein	Q99JF8	0.030	0.429	-2.331	6h + down vs 6h -
Alpha-galactosidase A	P51569	0.012	0.429	-2.329	6h + down vs 6h -
Nesprin-1	Q6ZWR6	0.026	0.430	-2.326	6h + down vs 6h -
Interleukin-6 receptor subunit beta	Q00560	0.038	0.433	-2.311	6h + down vs 6h -
Collagen alpha-1(V) chain	O88207	0.035	0.433	-2.309	6h + down vs 6h -
DNA-directed RNA polymerase II subunit RPB2	Q8CFI7	0.029	0.435	-2.300	6h + down vs 6h -
Actin-related protein 3	Q99JY9	0.038	0.435	-2.297	6h + down vs 6h -
Transcription elongation factor SPT5	O55201	0.049	0.436	-2.293	6h + down vs 6h -
Major vault protein	Q9EQK5	0.029	0.438	-2.284	6h + down vs 6h -
Aminoacyl tRNA synthase complex-interacting multifunctional protein 1	P31230	0.049	0.439	-2.278	6h + down vs 6h -
Sorting nexin-12	O70493	0.033	0.440	-2.273	6h + down vs 6h -
Lupus La protein homolog	P32067	0.044	0.441	-2.266	6h + down vs 6h -
Protein disulfide-isomerase TMX3	Q8BXZ1	0.047	0.442	-2.264	6h + down vs 6h -
COMM domain-containing protein 7	Q8BG94	0.034	0.445	-2.249	6h + down vs 6h -
Torsin-1A-interacting protein 1	Q921T2	0.048	0.446	-2.243	6h + down vs 6h -
Proteasome adapter and scaffold protein ECM29	Q6PDI5	0.035	0.447	-2.239	6h + down vs 6h -
Programmed cell death 6-interacting protein	Q9WU78	0.019	0.447	-2.239	6h + down vs 6h -
26S proteasome non-ATPase regulatory subunit 12	Q9D8W5	0.035	0.447	-2.238	6h + down vs 6h -
Inter-alpha-trypsin inhibitor heavy chain H2	Q61703	0.017	0.448	-2.234	6h + down vs 6h -
Proteasome subunit alpha type-4	Q9R1P0	0.042	0.448	-2.231	6h + down vs 6h -
Lysine--tRNA ligase	Q99MN1	0.006	0.448	-2.231	6h + down vs 6h -
T-complex protein 1 subunit eta	P80313	0.014	0.449	-2.228	6h + down vs 6h -
Nuclear pore glycoprotein p62	Q63850	0.028	0.450	-2.223	6h + down vs 6h -
TAR DNA-binding protein 43	Q921F2	0.046	0.450	-2.222	6h + down vs 6h -
Methylosome protein 50	Q99J09	0.023	0.453	-2.208	6h + down vs 6h -
Calcium/calmodulin-dependent protein kinase type II subunit gamma	Q923T9	0.024	0.453	-2.207	6h + down vs 6h -
Talin-1	P26039	0.009	0.455	-2.197	6h + down vs 6h -
Heterogeneous nuclear ribonucleoprotein M	Q9D0E1	0.044	0.455	-2.196	6h + down vs 6h -
NHL repeat-containing protein 2	Q8BZW8	0.044	0.456	-2.191	6h + down vs 6h -
Vasodilator-stimulated phosphoprotein	P70460	0.045	0.457	-2.189	6h + down vs 6h -
[Pyruvate dehydrogenase (acetyl-transferring)] kinase isozyme 3, mitochondrial	Q922H2	0.034	0.459	-2.178	6h + down vs 6h -
Eukaryotic initiation factor 4A-II	P10630	0.019	0.469	-2.134	6h + down vs 6h -
Heterogeneous nuclear ribonucleoprotein F	Q9Z2X1	0.045	0.471	-2.122	6h + down vs 6h -
Brain-specific angiogenesis inhibitor 1-associated protein 2	Q8BKX1	0.016	0.474	-2.109	6h + down vs 6h -
Glutamine-rich protein 1	Q3UA37	0.002	0.474	-2.108	6h + down vs 6h -
N-terminal kinase-like protein	Q9EQC5	0.047	0.475	-2.105	6h + down vs 6h -
ATP-citrate synthase	Q91V92	0.042	0.476	-2.099	6h + down vs 6h -
Phosducin-like protein 3	Q8BVF2	0.039	0.478	-2.093	6h + down vs 6h -
Cytochrome b-c1 complex subunit Rieske, mitochondrial	Q9CR68	0.036	0.480	-2.085	6h + down vs 6h -
Ectonucleoside triphosphate diphosphohydrolase 5	Q9WUZ9	0.038	0.484	-2.068	6h + down vs 6h -
14-3-3 protein eta	P68510	0.039	0.484	-2.067	6h + down vs 6h -
60S ribosomal protein L8	P62918	0.008	0.485	-2.062	6h + down vs 6h -
AH receptor-interacting protein	O08915	0.023	0.487	-2.055	6h + down vs 6h -
Probable ATP-dependent RNA helicase DDX5	Q61656	0.036	0.487	-2.053	6h + down vs 6h -

Dymeclin	Q8CHY3	0.034	0.488	-2.049	6h + down vs 6h -
Zinc finger CCCH-type antiviral protein 1	Q3UPF5	0.036	0.488	-2.048	6h + down vs 6h -
Transformer-2 protein homolog beta	P62996	0.030	0.490	-2.040	6h + down vs 6h -
Lamina-associated polypeptide 2, isoforms alpha/zeta	Q61033	0.034	0.493	-2.028	6h + down vs 6h -
Mitotic checkpoint protein BUB3	Q9WVA3	0.036	0.494	-2.023	6h + down vs 6h -
Nodal modulator 1	Q6GQT9	0.006	0.495	-2.021	6h + down vs 6h -
Importin-5	Q8BKC5	0.039	0.495	-2.021	6h + down vs 6h -
Adenylate kinase 2, mitochondrial	Q9WTP6	0.021	0.496	-2.018	6h + down vs 6h -
2-oxoglutarate dehydrogenase, mitochondrial	Q60597	0.026	0.496	-2.014	6h + down vs 6h -
Vam6/Vps39-like protein	Q8R5L3	0.015	0.496	-2.014	6h + down vs 6h -
Exportin-2	Q9ERK4	0.015	0.500	-1.999	6h + down vs 6h -
Thimet oligopeptidase	Q8C1A5	0.026	0.501	-1.997	6h + down vs 6h -
Serine/threonine-protein phosphatase 6 catalytic subunit	Q9CQR6	0.010	0.501	-1.996	6h + down vs 6h -
Transportin-1	Q8BFY9	0.021	0.502	-1.992	6h + down vs 6h -
Fascin	Q61553	0.002	0.503	-1.988	6h + down vs 6h -
Protein PBDC1	Q9D0B6	0.003	0.503	-1.986	6h + down vs 6h -
SRA stem-loop-interacting RNA-binding protein, mitochondrial	Q9D8T7	0.045	0.504	-1.985	6h + down vs 6h -
Serine/threonine-protein kinase DCLK1	Q9JLM8	0.031	0.504	-1.985	6h + down vs 6h -
Hexokinase-2	O08528	0.005	0.504	-1.984	6h + down vs 6h -
14-3-3 protein theta	P68254	0.042	0.505	-1.979	6h + down vs 6h -
Pre-B-cell leukemia transcription factor-interacting protein 1	Q3TVI8	0.012	0.507	-1.972	6h + down vs 6h -
Nucleosome assembly protein 1-like 1	P28656	0.032	0.512	-1.954	6h + down vs 6h -
Protein-L-isoaspartate(D-aspartate) O-methyltransferase	P23506	0.017	0.514	-1.945	6h + down vs 6h -
Biliverdin reductase A	Q9CY64	0.030	0.517	-1.934	6h + down vs 6h -
Signal transducer and transcription activator 6	P52633	0.039	0.517	-1.933	6h + down vs 6h -
Eukaryotic translation initiation factor 3 subunit E	P60229	0.032	0.518	-1.929	6h + down vs 6h -
Dual specificity mitogen-activated protein kinase kinase 3	O09110	0.001	0.519	-1.928	6h + down vs 6h -
Mitochondrial import inner membrane translocase subunit TIM50	Q9D880	0.048	0.520	-1.925	6h + down vs 6h -
Protein PRRC1	Q3UPH1	0.039	0.520	-1.924	6h + down vs 6h -
Proteasome subunit beta type-7	P70195	0.017	0.522	-1.917	6h + down vs 6h -
40S ribosomal protein S20	P60867	0.042	0.524	-1.910	6h + down vs 6h -
Prefoldin subunit 2	O70591	0.036	0.524	-1.908	6h + down vs 6h -
Stress-70 protein, mitochondrial	P38647	0.013	0.525	-1.905	6h + down vs 6h -
ADP-ribosylation factor-like protein 3	Q9WUL7	0.033	0.527	-1.897	6h + down vs 6h -
Histidine--tRNA ligase, cytoplasmic	Q61035	0.048	0.532	-1.881	6h + down vs 6h -
von Willebrand factor A domain-containing protein 8	Q8CC88	0.004	0.536	-1.864	6h + down vs 6h -
Tyrosine-protein phosphatase non-receptor type 23	Q6PB44	0.029	0.537	-1.864	6h + down vs 6h -
4-aminobutyrate aminotransferase, mitochondrial	P61922	0.041	0.538	-1.859	6h + down vs 6h -
Enoyl-[acyl-carrier-protein] reductase, mitochondrial	Q9DCS3	0.007	0.539	-1.856	6h + down vs 6h -
Protein FAM98B	Q80VD1	0.020	0.539	-1.855	6h + down vs 6h -
116 kDa U5 small nuclear ribonucleoprotein component	O08810	0.012	0.540	-1.852	6h + down vs 6h -
26S proteasome non-ATPase regulatory subunit 6	Q99JI4	0.041	0.540	-1.851	6h + down vs 6h -
28S ribosomal protein S31, mitochondrial	Q61733	0.012	0.541	-1.848	6h + down vs 6h -
Glutamate dehydrogenase 1, mitochondrial	P26443	0.008	0.542	-1.845	6h + down vs 6h -
Dolichyl-diphosphooligosaccharide--protein glycosyltransferase subunit 2	Q9DBG6	0.018	0.542	-1.844	6h + down vs 6h -
Cytochrome b-c1 complex subunit 1, mitochondrial	Q9CZ13	0.027	0.543	-1.843	6h + down vs 6h -
Quinone oxidoreductase	P47199	0.040	0.544	-1.839	6h + down vs 6h -
Integrator complex subunit 4	Q8CIM8	0.044	0.546	-1.833	6h + down vs 6h -
Importin subunit beta-1	P70168	0.046	0.546	-1.831	6h + down vs 6h -
Histone H1.0	P10922	0.007	0.546	-1.830	6h + down vs 6h -
Conserved oligomeric Golgi complex subunit 1	Q9Z160	0.008	0.548	-1.825	6h + down vs 6h -
OCIA domain-containing protein 2	Q9D8W7	0.033	0.548	-1.823	6h + down vs 6h -
AMP deaminase 3	O08739	0.026	0.550	-1.819	6h + down vs 6h -
Heme-binding protein 1	Q9R257	0.006	0.552	-1.812	6h + down vs 6h -
Thioredoxin-like protein 1	Q8CDN6	0.026	0.554	-1.805	6h + down vs 6h -
Very long-chain specific acyl-CoA dehydrogenase, mitochondrial	P50544	0.041	0.555	-1.802	6h + down vs 6h -
Vigilin	Q8VDJ3	0.022	0.557	-1.794	6h + down vs 6h -
26S proteasome non-ATPase regulatory subunit 8	Q9CX56	0.043	0.558	-1.794	6h + down vs 6h -
Plexin-B2	B2RXS4	0.014	0.558	-1.792	6h + down vs 6h -
Protein unc-45 homolog A	Q99KD5	0.035	0.560	-1.786	6h + down vs 6h -

cAMP-dependent protein kinase type II-beta regulatory subunit	P31324	0.049	0.560	-1.784	6h + down vs 6h -
S-methyl-5'-thioadenosine phosphorylase	Q9CQ65	0.039	0.563	-1.778	6h + down vs 6h -
Eukaryotic translation initiation factor 3 subunit F	Q9DCH4	0.032	0.564	-1.775	6h + down vs 6h -
Periodic tryptophan protein 1 homolog	Q99LL5	0.005	0.568	-1.760	6h + down vs 6h -
THO complex subunit 4	O08583	0.034	0.569	-1.759	6h + down vs 6h -
WASH complex subunit 2	Q6PGL7	0.004	0.571	-1.751	6h + down vs 6h -
Staphylococcal nuclease domain-containing protein 1	Q78PY7	0.043	0.587	-1.704	6h + down vs 6h -
NPC intracellular cholesterol transporter 2	Q9Z0J0	0.027	0.587	-1.703	6h + down vs 6h -
Acid sphingomyelinase-like phosphodiesterase 3b	P58242	0.037	0.588	-1.702	6h + down vs 6h -
Guanine nucleotide-binding protein G(I)/G(S)/G(O) subunit gamma-5	Q80SZ7	0.044	0.589	-1.699	6h + down vs 6h -
Haloacid dehalogenase-like hydrolase domain-containing protein 2	Q3UGR5	0.019	0.589	-1.697	6h + down vs 6h -
Importin subunit alpha-5	Q60960	0.041	0.592	-1.690	6h + down vs 6h -
Cleft lip and palate transmembrane protein 1 homolog	Q8VBZ3	0.018	0.594	-1.684	6h + down vs 6h -
Eukaryotic translation initiation factor 3 subunit B	Q8JZQ9	0.046	0.598	-1.671	6h + down vs 6h -
Glutathione S-transferase A4	P24472	0.014	0.605	-1.653	6h + down vs 6h -
Transcription factor BTF3	Q64152	0.047	0.605	-1.653	6h + down vs 6h -
DNA mismatch repair protein Msh6	P54276	0.045	0.609	-1.642	6h + down vs 6h -
Dihydrofolate reductase	P00375	0.021	0.610	-1.639	6h + down vs 6h -
Peptidyl-prolyl cis-trans isomerase FKBP2	P45878	0.020	0.613	-1.630	6h + down vs 6h -
Coiled-coil domain-containing protein 102A	Q3TMW1	0.024	0.618	-1.619	6h + down vs 6h -
39S ribosomal protein L13, mitochondrial	Q9D1P0	0.009	0.624	-1.602	6h + down vs 6h -
UHRF1-binding protein 1-like	A2RSJ4	0.050	0.625	-1.601	6h + down vs 6h -
CCR4-NOT transcription complex subunit 11	Q9CWN7	0.050	0.630	-1.587	6h + down vs 6h -
Mitochondrial import inner membrane translocase subunit TIM44	O35857	0.034	0.649	-1.541	6h + down vs 6h -
Dynein assembly factor 5, axonemal	B9EJR8	0.048	0.653	-1.531	6h + down vs 6h -
Alpha-1,3/1,6-mannosyltransferase ALG2	Q9DBE8	0.040	0.655	-1.527	6h + down vs 6h -
2-amino-3-ketobutyrate coenzyme A ligase, mitochondrial	O88986	0.041	0.663	-1.508	6h + down vs 6h -
Biogenesis of lysosome-related organelles complex 1 subunit 4	Q8VED2	0.043	0.671	-1.490	6h + down vs 6h -
DDRGK domain-containing protein 1	Q80WW9	0.011	0.671	-1.490	6h + down vs 6h -
CTP synthase 1	P70698	0.044	0.674	-1.483	6h + down vs 6h -
Heterogeneous nuclear ribonucleoprotein H2	P70333	0.000	0.684	-1.462	6h + down vs 6h -
Methylthioribulose-1-phosphate dehydratase	Q9WVQ5	0.011	0.691	-1.446	6h + down vs 6h -
39S ribosomal protein L39, mitochondrial	Q9JKF7	0.014	0.698	-1.432	6h + down vs 6h -
Peroxisomal membrane protein PMP34	O70579	0.043	0.701	-1.427	6h + down vs 6h -
Pyroglutamyl-peptidase 1	Q9ESW8	0.032	0.702	-1.424	6h + down vs 6h -
Actin-related protein 2/3 complex subunit 5	Q9CPW4	0.015	0.734	-1.363	6h + down vs 6h -
Nucleolysin TIAR	P70318	0.035	0.752	-1.330	6h + down vs 6h -
Peroxisomal acyl-coenzyme A oxidase 3	Q9EPL9	0.007	0.767	-1.304	6h + down vs 6h -
Alpha-2-macroglobulin receptor-associated protein	P55302	0.039	0.768	-1.302	6h + down vs 6h -
Anaphase-promoting complex subunit 13	Q8R034	0.012	0.777	-1.287	6h + down vs 6h -
NFU1 iron-sulfur cluster scaffold homolog, mitochondrial	Q9QZ23	0.032	0.778	-1.285	6h + down vs 6h -
Serine/threonine-protein kinase 24	Q99KH8	0.038	0.797	-1.254	6h + down vs 6h -
U5 small nuclear ribonucleoprotein 40 kDa protein	Q6PE01	0.010	0.806	-1.241	6h + down vs 6h -
Putative hydroxypyruvate isomerase	Q8R1F5	0.047	1.188	1.188	6h + up vs 6h -
Protein LSM12 homolog	Q9D0R8	0.006	1.312	1.312	6h + up vs 6h -
Vesicle-associated membrane protein 7	P70280	0.041	1.318	1.318	6h + up vs 6h -
Mixed lineage kinase domain-like protein	Q9D2Y4	0.029	1.359	1.359	6h + up vs 6h -
Methionine adenosyltransferase 2 subunit beta	Q99LB6	0.022	1.377	1.377	6h + up vs 6h -
Peroxisomal membrane protein PEX14	Q9R0A0	0.021	1.444	1.444	6h + up vs 6h -
Protein mono-ADP-ribosyltransferase PARP4	E9PYK3	0.043	1.472	1.472	6h + up vs 6h -
Sorbin and SH3 domain-containing protein 1	Q62417	0.042	1.534	1.534	6h + up vs 6h -
Protein ABHD16A	Q9Z1Q2	0.039	1.566	1.566	6h + up vs 6h -
Cytochrome c oxidase subunit 7A2, mitochondrial	P48771	0.022	1.584	1.584	6h + up vs 6h -
Core histone macro-H2A.1	Q9QZQ8	0.031	1.585	1.585	6h + up vs 6h -
U6 snRNA-associated Sm-like protein LSm1	Q8VC85	0.043	1.589	1.589	6h + up vs 6h -
Protein FAM98A	Q3TJZ6	0.010	1.703	1.703	6h + up vs 6h -
Ras-related protein Rab-10	P61027	0.048	1.791	1.791	6h + up vs 6h -
N-acetylgalactosamine kinase	Q68FH4	0.031	1.835	1.835	6h + up vs 6h -
Uracil phosphoribosyltransferase homolog	B1AVZ0	0.014	2.071	2.071	6h + up vs 6h -
Cadherin-11	P55288	0.014	2.173	2.173	6h + up vs 6h -
ATPase family AAA domain-containing protein 3	Q925I1	0.033	2.258	2.258	6h + up vs 6h -
CREB-regulated transcription coactivator 1	Q68ED7	0.032	2.322	2.322	6h + up vs 6h -

Huntingtin	P42859	0.018	2.447	2.447	6h + up vs 6h -
Proteolipid protein 2	Q9R1Q7	0.049	2.500	2.500	6h + up vs 6h -
F-box-like/WD repeat-containing protein TBL1XR1	Q8BHJ5	0.036	2.513	2.513	6h + up vs 6h -
Erlin-1	Q91X78	0.046	2.532	2.532	6h + up vs 6h -
ATP synthase subunit g, mitochondrial	Q9CPQ8	0.050	2.817	2.817	6h + up vs 6h -
Growth arrest-specific protein 1	Q01721	0.014	3.677	3.677	6h + up vs 6h -
NADH dehydrogenase [ubiquinone] 1 beta subcomplex subunit 9	Q9CQJ8	0.009	4.858	4.858	6h + up vs 6h -
